# Sorafenib tosylate inhibits directly necrosome complex formation and protects in mouse models of inflammation and tissue injury

**DOI:** 10.1038/cddis.2017.298

**Published:** 2017-06-29

**Authors:** Sofie Martens, Manhyung Jeong, Wulf Tonnus, Friederike Feldmann, Sam Hofmans, Vera Goossens, Nozomi Takahashi, Jan Hinrich Bräsen, Eun-Woo Lee, Pieter Van der Veken, Jurgen Joossens, Koen Augustyns, Simone Fulda, Andreas Linkermann, Jaewhan Song, Peter Vandenabeele

**Affiliations:** 1 VIB-UGent Center for Inflammation Research (IRC), Ghent, Belgium; 2 Department of Biomedical Molecular Biology (DBMB), Ghent University, Ghent, Belgium; 3 Department of Biochemistry, College of Life Science and Biotechnology, Yonsei University, Seoul, Korea; 4 Department of Internal Medicine III, Division of Nephrology, University Hospital Carl Gustav Carus at Technische Universität Dresden, Dresden, Germany; 5 Institute for Experimental Cancer Research in Pediatrics, Goethe-University, Frankfurt, Germany; 6 Laboratory of Medicinal Chemistry, University of Antwerp, Antwerp, Belgium; 7 Department of Pathology, Hannover Medical School, Hannover, Germany; 8 Metabolic Regulation Research Center, Korea Research Institute of Bioscience and Biotechnology (KRIBB), Daejeon, South Korea; 9 German Cancer Research Center (DKFZ), Heidelberg, Germany; 10 German Cancer Consortium (DKTK), Partner Site Frankfurt, Germany

## Abstract

Necroptosis contributes to the pathophysiology of several inflammatory, infectious and degenerative disorders. TNF-induced necroptosis involves activation of the receptor-interacting protein kinases 1 and 3 (RIPK1/3) in a necrosome complex, eventually leading to the phosphorylation and relocation of mixed lineage kinase domain like protein (MLKL). Using a high-content screening of small compounds and FDA-approved drug libraries, we identified the anti-cancer drug Sorafenib tosylate as a potent inhibitor of TNF-dependent necroptosis. Interestingly, Sorafenib has a dual activity spectrum depending on its concentration. In murine and human cell lines it induces cell death, while at lower concentrations it inhibits necroptosis, without affecting NF-*κ*B activation. Pull down experiments with biotinylated Sorafenib show that it binds independently RIPK1, RIPK3 and MLKL. Moreover, it inhibits RIPK1 and RIPK3 kinase activity. *In vivo* Sorafenib protects against TNF-induced systemic inflammatory response syndrome (SIRS) and renal ischemia–reperfusion injury (IRI). Altogether, we show that Sorafenib can, next to the reported Braf/Mek/Erk and VEGFR pathways, also target the necroptotic pathway and that it can protect in an acute inflammatory RIPK1/3-mediated pathology.

A delicate balance between cell death and proliferation is essential for tissue homeostasis in multicellular organisms.^1, 2^ Disturbance of this balance underlies the pathogenesis of various diseases, such as inflammatory and degenerative diseases, infectious diseases and cancer.^3^ Necrotic cell death is characterized by swelling and bursting of the cell, thereby releasing cytokines, chemokines and damage-associated molecular pattern molecules (DAMPs), which in a concerted way propagate inflammation.^4^ The discovery of necroptosis as a programmed form of necrosis that is regulated by the signaling of receptor-interacting protein kinases 1 and 3 (RIPK1/3),^5, 6, 7, 8, 9^ allowed to envisage necroptosis as a druggable process. Necroptosis can be triggered by DNA damage, immune receptors, viruses or death receptors of the TNF superfamily, such as Fas receptor (FasR), TRAILR1/2 or death receptor 3 (DR3),^2, 4^ but the best characterized is TNFR1-induced necroptotic signaling. Upon stimulation with TNF, TNF receptor 1 (TNFR1) trimerizes^10^ and a membrane-associated protein complex (TNFR1 complex I) is formed.^11^ Ubiquitylation of RIPK1 in this survival signaling complex results in activation of the I*κ*B kinase (IKK)-complex,^12^ which causes degradation of I*κ*B-*α* and thus NF-*κ*B activation. When inhibitors of apoptosis (IAPs), TGF*β* activated kinase-1 (TAK1) or inhibitor *κ*B kinases (IKKs) are blocked, RIPK1 kinase is activated and results in RIPK1-dependent apoptosis or necroptosis.^13^ When caspase-8 is inhibited^14, 15, 16^ or FADD is absent,^17^ the necrosome complex consisting of RIPK1 and RIPK3 is formed. Activated RIPK3 phosphorylates mixed lineage kinase domain like protein (MLKL), which relocates to the plasma membrane and directly^18, 19^ or indirectly^20, 21^ affects plasma membrane permeabilization. Necrostatins were the first identified inhibitors of necroptosis targeting RIPK1,^5, 22^ but no necrostatin-like drugs have been reported to have reached preclinical development. We identified the FDA-approved Sorafenib tosylate (further referred to as Sorafenib) as a potent inhibitor of necroptosis in two independent screening assays using L929 cells. Sorafenib is a multi-kinase inhibitor that induces apoptosis of cancer cells^23, 24, 25^ and is clinically used to treat advanced hepatocellular carcinoma (HCC), advanced renal cell carcinoma,^26^ and acute myeloid leukemia (AML).^27^ Sorafenib exerts anti-tumor effects by inhibiting kinases involved in cell proliferation and survival. It inhibits tumor cell proliferation^26, 28^ and angiogenesis^26^ through inhibition of Raf1 kinase^29^ and VEGFR1/2/3, PDGFRb, FGFR1 receptor tyrosine kinases^29^ respectively. Other cellular processes that are affected by Sorafenib are ER-stress mediated cell death independent of MEK and ERK^30^ and mTOR-mediated autophagy in HCC.^31^ It seems paradoxical that Sorafenib, a potent cytostatic and cytotoxic drug, inhibits necroptosis. We demonstrate that Sorafenib exerts this dual activity depending on its concentration. Furthermore, we show the inhibition of RIPK1 and RIPK3 as the mechanism underlying this novel function of Sorafenib.

## Results

### Compound screenings in cellular models of TNF-mediated necroptosis identify Sorafenib as a potent necroptosis inhibitor

**Figure 1:**
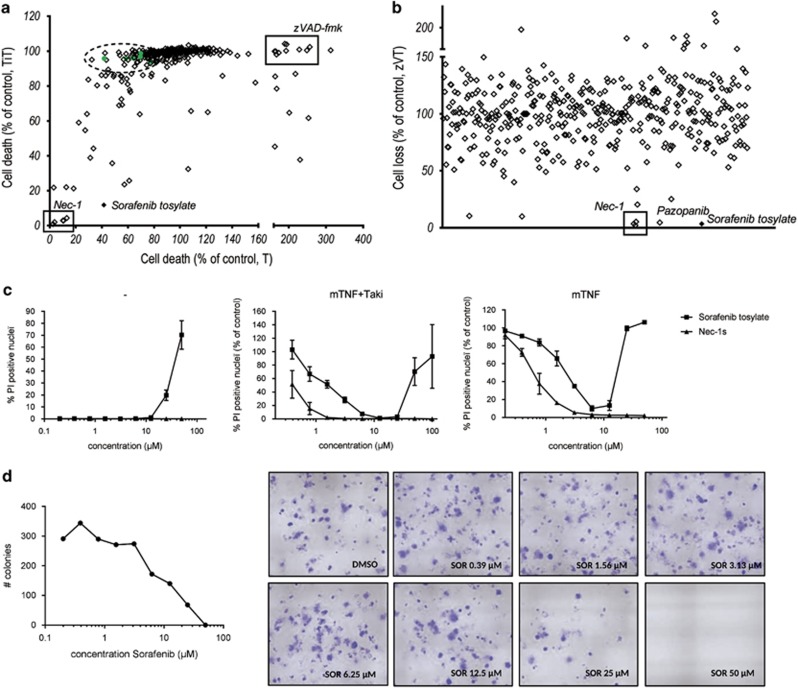
Compound screenings in cell models of TNF-mediated necroptosis identify Sorafenib as a potent necroptosis inhibitor, when used at non-cytotoxic concentrations. After compound treatment, L929sAhFas (**a**) or L929 (**b**) cells were stimulated with mTNF (T), mTNF+Tak1i (TiT) or mTNF+zVAD (zVT) (Supplementary Figure 1A and B). (**a**) Cell death percentage (% PI-positive nuclei) or cell loss was calculated as percent of control (DMSO+stimulus). Green points marked by the green dotted line represent a selection of kinase inhibitors from the screening on L929sAhFas cells (Supplementary Table 1), that are only slightly protective in the TNF-stimulated condition. (**b**) Cell loss (luminescence-based readout for ATP) was calculated as percent of control (DMSO+stimulus). (**c**) L929sAhFas cells were pretreated with DMSO, Nec-1s or Sorafenib (concentrations as indicated) for 19 h (mTNF+Tak1i) or 1 h (mTNF), followed by stimulation with 3.8 ng/ml mTNF+1 *μ*M Tak1i (mTTi) for 3 h, 0.2 ng/ml mTNF for 24 h or left unstimulated to determine cytotoxicity of the compound itself. In stimulated conditions, % PI-positive nuclei was determined as % of DMSO control. Data represent mean values±S.E.M. of two independent experiments. Cells were stained with Hoechst and PI and percentage PI-positive nuclei was determined using high-content image analysis (BD pathway Bioimager). (**d**) L929sAhFas cells were treated with Sorafenib (concentration as indicated) for 24 h. Then, stimulus was removed, 50 cells/condition seeded and colonies counted with crystal violet staining after 10 days incubation. Quantification was performed using ImageJ. *n*=2, a representative experiment is shown

To identify novel inhibitors or regulators of necroptosis, 500 small compounds from libraries targeting autophagy, kinases, phosphatases, proteases and redox balance were screened for their ability to block TNF-induced necroptosis in murine L929sAhFas cells. Three conditions were used to induce necroptosis in L929 cells: TNF alone, TNF+TAK1 inhibitor (TAK1i) and TNF+zVAD-fmk, a pan-caspase inhibitor. Inhibition of TAK1 or caspases sensitizes to necroptosis.^13, 32^ Necroptosis, measured as the percentage of PI-positive nuclei normalized to the plate-specific DMSO control, was determined for each compound (Supplementary Figure 1A). Z’ values for the TNF- and TNF+TAK1i-induced cell death assay were 0.498 and 0.919, respectively (Supplementary Figures 2A and B), indicating good assay performance. Sorafenib was identified as an effective inhibitor of both TNF- and TNF+TAK1i-induced necroptosis (Figure 1a). Sorafenib reduced TNF-induced cell death to 40% of control, and it almost completely blocked TNF+TAK1i-induced cell death. In an independent screening with 437 FDA-approved drugs and TNF+zVAD-fmk as necroptosis trigger in L929 cells (Supplementary Figure 1B), Sorafenib was also shown to be a potent inhibitor (Figure 1b). Under sensitizing conditions (TAK1i or zVAD-fmk), Sorafenib (10 *μ*M) was equally potent as necrostatin-1 (10 *μ*M) and the recently reported RIPK1 inhibitor pazopanib, a multi-target inhibitor of VEGFR1/2/3, PDGFR, FGFR, c-KIT and c-Fms.^33^ Other kinase inhibitors from the screening with similar targeting profiles as Sorafenib did not protect against necroptosis (Supplementary Tables 1–3), suggesting that necroptosis inhibition by Sorafenib is not due to inhibition of its known targets (Raf1 kinase, VEGFR1/2/3, PDGFRb, FGFR1 receptor tyrosine kinases).^29^

### Sorafenib protects against TNF-induced cell death at non-toxic concentrations

Since Sorafenib induces apoptosis and cytostasis,^29, 33^ we determined dose-responses in the presence or absence of necroptosis stimulus. As a chemotherapeutic drug Sorafenib induces cell death in a dose- and time-dependent manner at concentrations above 25 *μ*M, reaching close to 100% cell death after 48 h at 100 *μ*M in L929sAhFas cells (EC50 is 39.6 *μ*M) (Figure 1c, left panel; Supplementary Figure 3). A dose-dependent cytostatic effect of Sorafenib was observed 10 days after removal of stimulus in a clonogenic assay (Figure 1d). However, in the same L929sAhFas cells, Sorafenib inhibited necroptosis induced by TNF alone or in sensitized condition (TNF+TAK1i) at concentrations below 10 *μ*M without inducing cell death (Figure 1c, right panel). Altogether, these data demonstrate that Sorafenib is cytotoxic at high concentration on its own, while it protects against TNF-induced necroptosis at more than 10-fold lower concentration (Figure 1c; Supplementary Table 4).

### Sorafenib inhibits TNF-induced RIPK1-dependent cell death in murine and human cell lines

**Figure 2:**
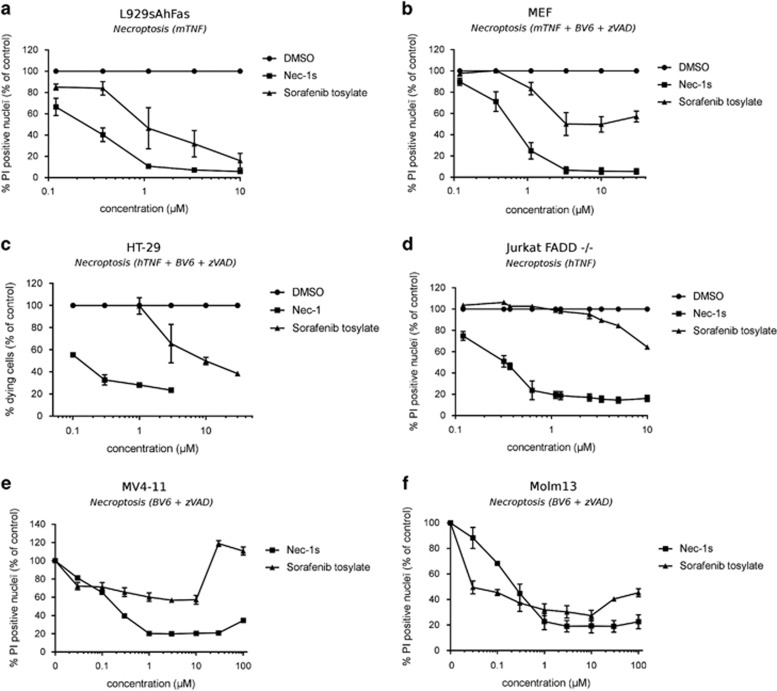
Sorafenib inhibits TNF-induced RIPK1-dependent cell death in both murine and human cell lines and rescues AML cells from Smac mimetic-induced necroptosis. (**a**,**b**) L929sAhFas and MEF cells were pretreated for 1 h with DMSO, Nec-1s or Sorafenib (concentration as indicated) and stimulated for 4.25 h (L929) or 3 h (MEF) with 38 ng/ml mTNF, 1 *μ*M BV6 and 10 *μ*M zVAD, as indicated. (**c**,**d**) HT-29 and Jurkat FADD−/− cells were pretreated for 1 h with DMSO, Nec-1 or Sorafenib (concentrations as indicated) and stimulated for 6 h with 30 ng/ml hTNF, 1 *μ*M BV6 and 30 *μ*M zVAD (HT-29) or stimulated for 10 h with 100 ng/ml hTNF (Jurkat FADD−/−). (**a**-**d**) Data were normalized to DMSO-treated control cells and represent the mean value±S.E.M. of three independent experiments. Toxic concentrations were removed from the analysis. L929sAhFas and MEF cells were stained with Hoechst and PI and percentage PI-positive nuclei was determined using high-content image analysis (BD pathway Bioimager). For HT-29 and Jurkat FADD−/− cells, cell death was determined by PI staining and flow cytometry. (**e**,**f**) MV4-11 and Molm13 cells were pretreated for 1h with 40 *μ*M zVAD-fmk and Sorafenib or Nec-1s (concentration as indicated) and stimulated for 6h (Molm13) or 24h (MV4-11) with 3 *μ*M BV6. Cell death in AML cells was determined by PI staining and flow cytometry. Mean and S.D. of three independent experiments performed in triplicate are shown

To confirm the inhibition of TNF-induced necroptosis by Sorafenib, we tested a panel of murine (L929sAhFas and MEF) and human cell lines (HT-29, Jurkat FADD^-/-^, and two AML cell lines MV4-11 and Molm13). Smac mimetic BV6^34^ instead of TAK1i was used as a sensitizer for testing of mouse embryonic fibroblasts (MEF) and human cell lines. Dose-response curves and IC50 values for the potency of Sorafenib to inhibit cell death, were determined and compared with Nec-1s, as a reference compound.^22^ As for murine cells, L929sAhFas and MEF cells stimulated with mTNF or with mTNF/smac mimetic BV6/zVAD-fmk (Figures 2a and b), Sorafenib inhibited necroptosis with an IC50 value of 1.27 *μ*M and 3.48 *μ*M respectively, as compared with 0.24 *μ*M and 0.63 *μ*M for Nec-1s (Figures 2a and b; Supplementary Table 4). To investigate whether the protective effect of Sorafenib at <10 *μ*M is specific for necroptosis, we tested apoptotic conditions in both L929sAhFas and MEF cells. Pretreatment with Sorafenib did not protect L929sAhFas cells from apoptosis induced by agonistic anti-Fas antibody (AF). However, Sorafenib did protect against RIPK1-dependent apoptosis in MEF cells induced by mTNF/BV6 (IC50 of 2.20 *μ*M) (Supplementary Figure 4B). This demonstrates that Sorafenib inhibits RIPK1-dependent apoptosis and necroptosis equally, but not RIPK1-independent apoptosis. As for human cell lines, Sorafenib protected against necroptotic stimuli in HT-29 cells (Figure 2c) as well as Jurkat FADD^-/-^ cells (Figure 2d). Since Sorafenib is used to treat AML,^27^ we also included two AML cell lines to investigate whether it alters the sensitivity of these cells to necroptotic stimuli (BV6 in the presence of zVAD-fmk).^34^ Sorafenib significantly reduced BV6/zVAD-fmk-induced necroptosis of AML cells in a dose-dependent manner (Figures 2e and f). Similar to the L929sAhFas cell line (Figure 1c), the human AML cells underwent Sorafenib-induced cell death (EC50 of 19.6 *μ*M for MV4-11 cells) (Supplementary Figures 4C and D), while they were protected against BV6+zVAD (IC50 Molm13 of⩽0.03 *μ*M) (Figures 2e and f). Addition of Nec-1s significantly decreased BV6/zVAD-fmk-mediated necroptosis of AML cells (Figures 2e and f). In all human cell lines tested Sorafenib protected against necroptosis stimuli (Figures 2c–f) but with variable IC50 values (Supplementary Table 4). Overall, we confirm that Sorafenib at <10 *μ*M protects against TNF-induced RIPK1-dependent cell death, with a three-fold higher efficiency in murine cell lines than in human cell lines.

### Sorafenib does not influence the TNF-induced NF-*κ*B activation, but protects against necroptosis by targeting the necrosome complex

**Figure 3:**
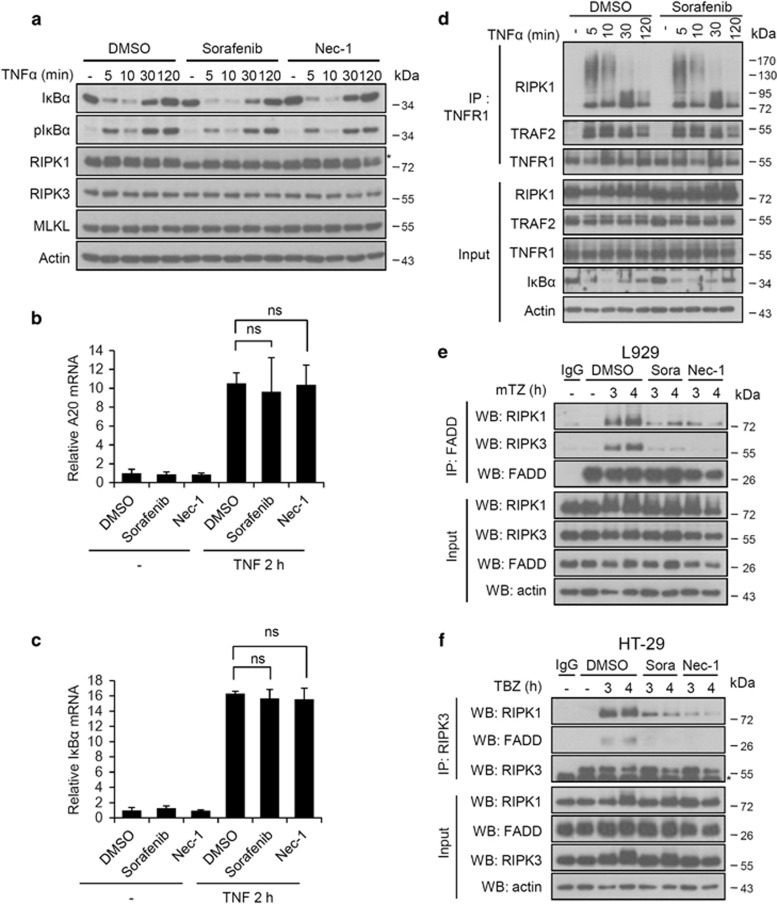
Sorafenib does not influence complex I formation, but protects against necroptosis by targeting the necrosome complex. (**a**) L929 cells were pretreated with 10 *μ*M Nec-1s, Sorafenib or DMSO for 1 h and stimulated with mTNF (10 ng/ml) for the indicated time. Cells were lysed and immunoblotted with the indicated antibodies. *Phosphorylated RIPK1. (**b**,**c**) L929 cells were pretreated with 10 *μ*M DMSO, Nec-1 or Sorafenib for 1 h and stimulated with mTNF (10 ng/ml) for 2 h. The relative mRNA levels of mA20 and mI*κ*B-*α* were analyzed by qRT-PCR. All bars represent mean±S.D.; *n*=3, ns=non-significant. (**d**) L929 cells were pretreated with 10 *μ*M Sorafenib or DMSO and stimulated with mTNF (10 ng/ml) for the indicated time. Cell lysates were immunoprecipitated with anti-mTNFR1 antibody. (**e**,**f**) L929 (**e**) and HT-29 (**f**) cells were pretreated with 10 *μ*M Nec-1, Sorafenib (Sora) or DMSO and stimulated with zVAD (10 *μ*M)+mTNF (5 ng/ml) (L929) or zVAD (30 *μ*M)+BV6 (1 *μ*M)+hTNF (30 ng/ml) (HT-29) for the time indicated. Cell lysates were immunoprecipitated with the indicated antibodies and both immunoprecipitates (IP) and total lysates (input) were immunoblotted with the indicated antibodies (WB)

We questioned whether NF-*κ*B mediated survival signaling was involved in the protection by Sorafenib. Similar to Nec-1s, Sorafenib pretreatment did not prevent the phosphorylation and degradation of I*κ*B-*α*, the inhibitor of NF-*κ*B,^35^ after TNF stimulation of L929 or L929sAhFas cells (Figure 3a; Supplementary Figure 5). We also examined relative mRNA levels of NF-*κ*B responsive genes, including A20 and I*κ*B-*α*.^36^ TNF stimulation of L929 cells (Figures 3b and c) and L929sAhFas cells (Supplementary Figure 6) resulted in a ten-fold induction of A20 and I*κ*B-*α* mRNA, and the presence of Sorafenib did not affect this gene induction. Thus NF-*κ*B activation after TNF stimulation and subsequent survival signaling is not impaired by Sorafenib. Moreover, Sorafenib did not affect the RIPK1, RIPK3 and MLKL protein levels (Figure 3a), excluding their transcriptional control as a mechanism of Sorafenib-mediated inhibition of necroptosis. Although A20 and I*κ*B-*α* gene induction was not affected, induction of cytokines and chemokines (TNF-*α*, MIP-2, MCP1 and CXCL1) was strongly reduced by Sorafenib (Supplementary Figure 6), suggesting that other kinases involved in transcriptional regulation are affected by Sorafenib. The above results suggested that the formation of complex I and RIPK1 polyubiquitylation, absolute requirements for NF-*κ*B signaling, are not impaired by Sorafenib. Indeed, immunoprecipitation of TNFRI after mTNF*α* stimulation showed similar patterns of RIPK1 polyubiquitylation in both DMSO- and Sorafenib-pretreated L929 cells (Figure 3d). Immunoprecipitation of FLAG-hTNF after 5 min of stimulation of L929sAhFas cells resulted in polyubiquitylation of RIPK1, which was not altered by Nec-1s or Sorafenib treatment (Supplementary Figure 7). Next, we investigated whether necrosome formation, involving activation and autophosphorylation of both RIPK1 and RIPK3,^5, 6, 7, 8^ was affected by pretreatment with Sorafenib. The recruitment of both RIPK1 and RIPK3 to FADD after 3–4 h of TNF/zVAD-fmk stimulation of L929 cells was markedly reduced in Sorafenib-treated L929 cells (Figure 3e). Sorafenib not only inhibited necrosome formation in murine L929 cells, but also in human HT-29 cells (Figure 3f). Collectively, these data show that Sorafenib inhibits neither TNF complex I formation nor NF-*κ*B signaling, but interferes with necrosome formation preventing necroptosis to occur.

### Identification of RIPK1 and RIPK3 as targets of Sorafenib

**Figure 4:**
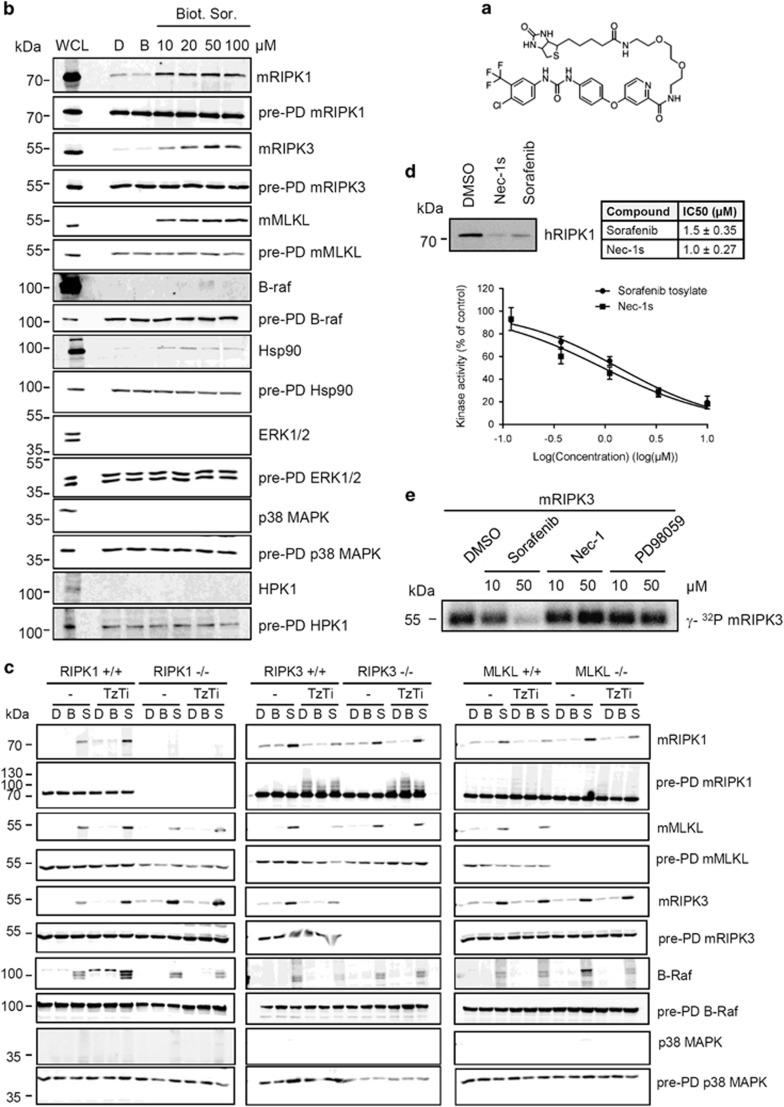
Identification of RIPK1 and RIPK3 as targets of Sorafenib tosylate. (**a**) Structure of biotinylated Sorafenib tosylate. (**b**,**c**) Streptavidin pull down of biotinylated Sorafenib. DMSO (D), biotin (**b**) or 10 *μ*M/20 *μ*M/50 *μ*M/100 *μ*M biotinylated Sorafenib (biot. Sor.) was added to L929sAhFas cell lysate and 50 *μ*M biotinylated Sorafenib (S) to MEF cell lysates, followed by pull down with streptavidin beads. Both pull down samples and total lysates (pre-PD) were analyzed by SDS-PAGE and immunoblotted with the indicated antibodies. L929sAhFas whole cell lysate (WCL) was included as loading control. (**d**) *In vitro* non-radioactive ATPγS kinase assay (upper figure) and *in vitro* ADP-Glo kinase assay (lower figure and table) using recombinant hRIPK1 protein (100 nM). Recombinant hRIPK1 was incubated with 50 *μ*M Nec-1s, Sorafenib or DMSO (upper figure) or with a concentration as indicated. (**e**) FLAG-mRIPK3 purified from 293FT cells was pre-incubated with indicated chemicals (conc. as indicated) for 15 min at 25 °C and incubated with [γ-^32^P]ATP for *in vitro* radioactive kinase assay

Since Sorafenib is a broad-spectrum tyrosine kinase inhibitor,^26^ it may target both RIP kinases and other kinases important for TNF cytotoxic signaling. Prevention of necrosome formation by Sorafenib implies that Sorafenib may interact with RIPK1 and/or RIPK3. To identify targets of Sorafenib during TNF signaling, biotinylated Sorafenib was synthesized (Figure 4a). Several Sorafenib variants were synthesized containing different substituents in the R-position (Supplementary Figure 8), to test whether biotinylation of Sorafenib was possible without losing its inhibitory potency. The biotinylated analog of Sorafenib showed a lower inhibitory potency and thus had to be used at higher concentrations for similar efficacy (Supplementary Figure 8, compound 10). To identify binding partners of biotinylated Sorafenib, a pull down of biotinylated Sorafenib in unstimulated cell lysates of L929sAhFas was performed. We show a dose-dependent pull down of RIPK1, RIPK3, MLKL and B-Raf (Figure 4b; Supplementary Figure 9). Other kinases, like ERK1/2, p38MAPK and HPK1, were not detected in the pull down, demonstrating the specificity of the binding. Detection of Hsp90 in the pull down is not suprising, as Hsp90 is known to act as a chaperone for RIPK1 stabilization,^37^ RIPK3 activation^38^ and MLKL oligomerization^39^ during necroptosis execution (Figure 4b). These data suggested that Sorafenib binds either to a (pre-formed) multi-protein complex containing RIPK1, RIPK3 and MLKL or to all three proteins independently. Interestingly, we were able to pull down each of the three necrosome components (RIPK1, RIPK3, MLKL) in MEF cell lysates from the following mice strains: RIPK1+/+, RIPK1−/−, RIPK3+/+, RIPK3−/−, MLKL+/+ and MLKL−/−. This experiment demonstrates that Sorafenib is likely to bind independently to RIPK1, RIPK3 or MLKL (Figure 4c), even in the absence of a necroptotic stimulus. Together with the data of the immunoprecipitation of TNFR1 (complex I) and FADD (complex II) after TNF stimulation, we conclude that Sorafenib can bind RIPK1, RIPK3 and MLKL and hereby prevents the formation of the necrosome.

In order to test whether Sorafenib directly inhibits kinase activities of RIPK1 or RIPK3, different *in vitro* kinase assays were performed (Figures 4d and e). A non-radioactive *in vitro* ATPγS kinase assay was performed with recombinant GST-hRIPK1.^40^ Incubation of recombinant GST-hRIPK1 with Nec-1s or Sorafenib resulted in a strong decrease in RIPK1 autophosphorylation compared with the DMSO control, although Sorafenib was less efficient than Nec-1s (Figure 4d, upper figure). IC50 values of Sorafenib and Nec-1s were 1.5 *μ*M and 1 *μ*M respectively in an *in vitro* ADP-Glo kinase assay^41^ using recombinant hRIPK1 (Figure 4d, lower figure), confirming the results of the *in vitro* ATPγS kinase assay. Finally, 50 *μ*M Sorafenib inhibited mRIPK3 autophosphorylation in a radioactive kinase assay with FLAG-mRIPK3 (Figure 4e). Although Sorafenib can bind to MLKL in cell lysate, it does not protect against ligand independent MLKL-induced cell death (Supplementary Figure 10), excluding functional targeting on MLKL. Altogether, these data illustrate that Sorafenib can bind to RIPK1 and RIPK3 and inhibit their kinase activities.

### Sorafenib protects against TNF-induced systemic inflammatory response syndrome and renal ischemia–reperfusion injury

**Figure 5:**
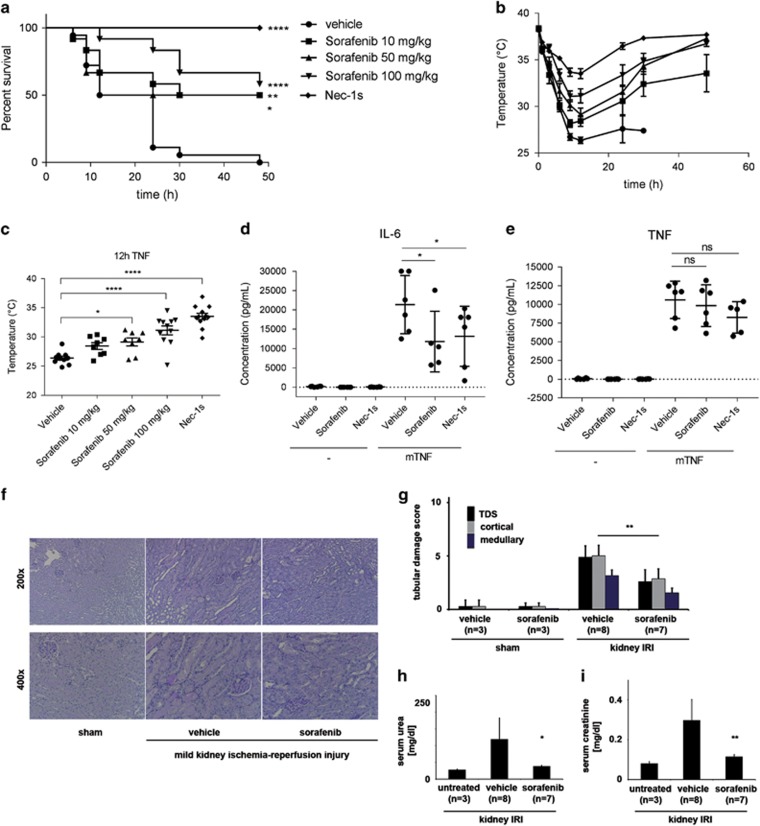
Sorafenib protects against TNF-induced systemic inflammatory response syndrome (SIRS) and renal ischemia–reperfusion injury (IRI). Survival curve (**a**) and body temperature (**b**,**c**) (means±S.E.M.) of WT mice (*n*=12) injected with mTNF (500 *μ*g/kg i.v.) after pretreatment with Nec-1s (6.25 mg/kg i.v.) or Sorafenib (10/50/100 mg/kg gavage). Control mice (*n*=12) received an equal amount of vehicle before the mTNF challenge. Results of three independent experiments are shown. (**d**,**e**) Plasma samples of Solvent-, Nec-1s- (6.25 mg/kg i.v.) and Sorafenib-treated mice (100 mg/kg gavage) (*n*=6/group) were collected 0 h and 6 h after mTNF challenge (375 *μ*g/kg i.v.) and analyzed for mTNF-*α* and mIL-6. (**c**–**e**) A one-way ANOVA test with Bonferroni multiple-testing correction was performed. *P<0.05, **P<0.01, and ****P<0.0001 *versus* control mice. (**f–i**) Histology, tubular damage score and serum urea/creatinine levels of mice treated with vehicle or Sorafenib (10 mg/kg i.p.) 15 min before initiation of ischemia. Mice were killed 48 h after reperfusion and kidneys were removed after retro-orbital blood puncture. Stained kidney sections were analyzed using an Axio Imager microscope (Zeiss). Organ damage was quantified by an experienced pathologist in a double-blind manner on a scale ranging from 0 (unaffected tissue) to 10 (severe organ damage), *P<0.05 and **P<0.01

To examine whether Sorafenib could protect against RIPK kinase-driven inflammation *in vivo*, we tested it in the TNF-induced SIRS model. This is a sterile model of sepsis that depends on RIPK1 kinase activity and RIPK3.^42, 43^ Sorafenib, administered by gavage 1.5 h before *i.v.* mTNF treatment, significantly protected mice from hypothermia and death caused by mTNF in a dose-dependent manner (Figures 5a and b). Mice pretreated with Nec-1s were fully protected, while about 50% of mice pretreated with Sorafenib survived (Figure 5a). The IL-6 concentration in plasma of Sorafenib-treated mice (100 mg/kg), like Nec-1s-treated mice, are significantly lower than vehicle-treated mice after 6 h TNF challenge (Figure 5d). On the other hand, TNF concentration was not significantly lower under these conditions (Figure 5e). In conclusion, these results indicate that Sorafenib not only protects against RIPK1/3-dependent cell death *in vitro*, but also against RIPK1/3-dependent lethality in TNF-induced SIRS. As Nec-1 treatment and RIPK3-deficiency are beneficial in the model of renal ischemia–reperfusion injury,^44, 45^ we investigated the effect of Sorafenib in this model of tissue injury and inflammation as well. Low-dose Sorafenib protected against histological damage (Figures 5f and g) and functionally ameliorated acute renal failure, as demonstrated by reduced serum creatinine and urea 48 h after reperfusion (Figures 5h and i). Higher dose of Sorafenib however sensitized the mice to ischemic damage and led to deterioration of acute renal failure (Supplementary Figure 11). We conclude that Sorafenib provided protection in two *in vivo* models of tissue injury and inflammation driven by RIPK1/RIPK3-dependent cell death.

## Discussion

We performed cellular screenings with targeted small compound libraries (for kinases, phosphatases, proteases, redox-regulation, autophagy) and an FDA-approved drug library in order to identify inhibitors of necroptosis. From the results, we selected Sorafenib for further characterization as an inhibitor of necroptosis, not only because it protected against different necroptosis inducing stimuli but also because it is an FDA-approved drug that is used in clinic to treat advanced hepatocellular carcinoma,^46^ renal cell carcinoma^47^ and AML.^27^ Several necroptosis inhibitors have been discovered or designed to target RIPK1,^5, 48^ RIPK3^49^ and MLKL,^50, 51^ the core necroptosis pathway,^52^ but none of them are already in clinical use.^53^ Sorafenib is not the only anti-cancer drug that can inhibit necroptosis. Recently, Pazopanib, first proposed as a multi-target inhibitor of VEGFR1/2/3, PDGFR, FGFR, c-KIT and c-Fms and approved for the treatment of advanced renal cell carcinoma, and Ponatinib, first described as Pan-Bcr-Abl tyrosine kinase inhibitor and approved for the treatment of chronic myeloid leukemia, were both reported as necroptosis inhibitors at the submicromolar range,^33^ the former blocking mainly RIPK1 and the second blocking both RIPK1 and RIPK3. Ponatinib has been used as template to design new inhibitors with improved selectivity for RIPK1.^54^ The B-Raf^V600E^ inhibitors Vemurafenib and Dabrafenib inhibit RIPK3, with Dabrafenib being effective in the submicromolar range.^55^ Dabrafenib competes with ATP for binding to RIPK3 and alleviates acetaminophen-induced liver injury.^55^ Here, we demonstrate that the anti-cancer agent Sorafenib acts as an inducer of cell death at high concentration but as an inhibitor of RIPK-dependent cell death at lower concentration.

The kinase domain of B-raf^V600E^ has strong structural homology with the kinase domains of hRIPK1 and hRIPK3, which explains the similarities between the crystal structure of Nec-1 bound to RIPK1 and the structure of Vemurafenib bound to B-Raf^V600E^.^56^ The crystal structure of B-Raf bound to Sorafenib indicates that Sorafenib occupies the ATP-binding pocket and keeps the protein in an inactive conformation.^57^ As the broad-spectrum tyrosine kinase inhibitor Sorafenib was originally designed as a Raf1-inhibitor,^26^ and given the homology between B-Raf^V600E^ and the kinase domain of hRIPK1/3,^56^ we speculated that Sorafenib could also bind RIPK1/3 by targeting the kinase domain. The protection of Sorafenib against TNF-induced necroptosis cannot be explained by inhibition of its known targets (Raf B/C kinase, VEGFR1/2/3, PDGFR, c-KIT, RET, Flt3),^58^ since small compounds with similar target profiles, like Vandetanib targeting VEGFR-2/KIT/PDGFR, do not protect from necroptotic cell death.^33^ Also, compounds like GW5074, SU1498, Tyrphostin AG1295 and PP1, targeting Raf kinase, VEGFR, PDGFR and Src kinase respectively do not inhibit necroptosis (Supplementary Tables 1–3). Moreover, we show that Sorafenib can bind either a complex containing RIPK1, RIPK3 and MLKL or to each of these proteins separately. Finally, we showed that Sorafenib protected against two models of RIPK-driven inflammation and tissue injury, namely TNF-induced SIRS, a sterile model for sepsis,^42^ and renal ischemia–reperfusion injury (IRI).^59^ It has been shown that Sorafenib can be used efficiently to block Hepatitis C virus replication and viral gene expression^60^ and Rift Valley fever virus infection.^61^ It is also reported that Sorafenib can protect against ischemia/reperfusion (IR) injury in rats with nonalcoholic steatohepatitis,^62^ is able to reverse LPS-induced hypotension by targeting soluble epoxide hydrolase (sEH), an enzyme with pleiotropic effects on inflammation and vascular disease,^63^ and is also able to restore working memory abilities in a mouse model for Alzheimer’s disease (AD) by inhibition of the pro-neuroinflammatory factor cRaf-1 and NF-*κ*B.^64^ Thus Sorafenib exerts an anti-inflammatory activity in other *in vivo* experimental diseases models. Given the inflammatory response triggered by release of cytokines, chemokines and DAMPs from necroptotic dying cells,^42, 59^ these reported anti-inflammatory effects of Sorafenib *in vivo* may be attributed to its potent necroptosis targeting effect we describe here. In HCC, chronic inflammation is known to promote tumor progression and metastasis, with a pivotal role for TNF in promoting invasion, angiogenesis and metastasis.^65, 66^ As necroptosis through DR6 in tumor-associated endothelial cells has been described as a metastasis promoting process,^67^ the efficacy of Sorafenib in HCC could, besides the direct cytotoxicity on tumor cells, be due to necroptosis inhibition preventing pro-inflammatory DAMP release in the necrotic core and so reducing the inflammation-dependent tumor growth^68^ and preventing endothelial necroptosis and metastasis.^67^ As Sorafenib is not specifically inhibiting necroptosis but also RIPK1 kinase dependent apoptosis, one could envisage that during experimental disease models *in vivo* it may target simultaneously RIPK1-dependent necroptosis and apoptosis. Recent evidence illustrates that TNF-induced SIRS is a consequence of both caspase-8-dependent apoptosis and MLKL-dependent necroptosis.^43^ Sorafenib inhibits both RIPK1-dependent apoptosis and necroptosis, which may explain its protective effect during TNF-induced SIRS model.

Repurposing FDA-approved drugs is a new and valuable strategy to offer new therapies for diseases that remain difficult to treat^69, 70^ such as sepsis, degenerative disorders and metabolic diseases. However, more extensive *in vivo* experiments need to be performed to explore possible repurposing of Sorafenib to treat these human disease conditions. Our data also indicate that Sorafenib at low concentrations can interfere with therapeutic induction of necroptotic cell death in AML cells. Since some targeted therapies such as Smac mimetic in combination with epigenetic drugs have been shown to trigger necroptosis as an alternative mode of cell death in apoptosis-resistant AML cells,^71, 72^ these findings imply that Sorafenib may limit the anti-leukemic activity under certain conditions. To conclude, we identified the necroptotic pathway as a novel Sorafenib-targeted pathway through inhibition of the necrosome formation. We also demonstrated that Sorafenib alleviated inflammation and tissue injury in two experimental disease models in mice which are driven by RIPK-dependent cell death, suggesting that necroptosis targeting could be part of the therapeutic potential of Sorafenib.

## Materials and methods

### Cell culture, cytokines and reagents

L929 cells were purchased from ATCC (used in Korean Lab), L929 cells stably transfected with hFas and designated as L929sAhFas (used in Belgian Lab) were generated as previously described.^14^ All L929 cell lines and MEF cells were cultured in DMEM supplemented with 10% (v/v) FCS and l-glutamine (0.03%). Jurkat FADD^−/^^−^ cells were cultured in RPMI medium supplemented with 15% (v/v) FCS, sodium pyruvate (400 *μ*M) and l-glutamine (0.03%). HT-29 cells were cultured in McCoy’s 5A medium supplemented with 10% (v/v) FBS. AML cell lines were obtained from DSMZ (Braunschweig, Germany) and were cultured in RPMI 1640 medium (Life Technologies, Eggenstein, Germany), supplemented with 10% FCS (Biochrom, Berlin, Germany), 1 mM Pyruvate (Invitrogen, Karlsruhe, Germany) and 1% penicillin/streptomycin (Invitrogen). Following cytokines and reagents were used: recombinant (rec) human and murine TNF were produced at VIB protein Service Facility (Ghent, Belgium) with a specific biological activity of 3 × 10^7^ IU/ml and 1.27 × 10^8^ IU/ml (batch 2: 4.93 × 10^7^ IU/ml) respectively, human and murine rec TNF were also purchased from Sigma-Aldrich (T0157) and eBioscience (14-8321-62) respectively (Korean Lab), rec FLAG-hTNF was produced at VIB Protein Service Facility (Ghent, Belgium) and was used at 1.5 *μ*g/ml. Following reagents were purchased as indicated: necrostatin-1 (Calbiochem, San Diego, CA, USA and Enzo life science, Farmingdale, NY, USA), necrostatin-1s (synthesized by the laboratory of Medicinal Chemistry; University of Antwerp), Sorafenib tosylate (Selleck Chemicals, Houston, TX, USA), SMAC mimetic BV6 (Selleck Chemicals), Tak1 kinase inhibitor NP-009245 (AnalytiCon Discovery GmbH, Potsdam, Germany), zVAD-fmk (Bachem, Bubendorf, Switzerland and R&D systems) and agonistic anti-human Fas (clone 2R2, Cell Diagnostica, Munster, Germany). Libraries used are: autophagy library (BML-2837), epigenetics library (BML-2836), kinase inhibitor library (BML-2832), phosphatase inhibitor library (BML-2834), protease inhibitor library (BML-2833), redox library (BML-2835) (Screen-well, Enzo Life Sciences) and 437 FDA-approved drug library (SelleckChem, L1300).

### Antibodies

Antibodies used were anti-RIPK1 (BD Biosciences, San Jose, CA, USA, #610459), anti-actin (MP Biomedicals, Solon, OH, USA, #69100), anti-I*κ*B-*α* (Santa Cruz Biotechnology, Dallas, TX, USA, #sc-371), anti-hRIPK3 (ThermoFisher Scientific Pierce, Waltham, MA, USA, PA1-41533), anti-mRIPK3 (Sigma-Aldrich, St Louis, MO, USA, #R4277 and IMGENEX, San Diego, CA, USA,, IMG-5523-2), anti-hMLKL (Genetex, Irvine, CA, USA, GTX107538), anti-mMLKL (Millipore, Billerica, MA, USA, #MABC604 and Abgent, San Diego, CA, USA, AP14272b-ev), anti-phospho-hMLKL (Abcam, Milton, Cambridge, UK, #187091), anti-hFADD (BD Biosciences, 610399), anti-mFADD (Millipore, 05-486), anti-thiophosphate ester (Epitomics, Burlingame, CA, USA, #2686-1), anti-Braf (ThermoFisher scientific, MA5-15495), anti-HPK1 (Cell Signaling, Danvers, MA, USA, #4472), anti-Hsp90 (Santa Cruz Biotechnology, sc-7947), anti-p38MAPK (Cell Signaling, #9212), anti-ERK1/2 (Cell Signaling, #9102), anti-Flag-HRP (Sigma-Aldrich, St Louis, MO, USA, #A8592) and anti-tubulin-HRP (Abcam, #ab21058). Secondary antibodies used were HRP-conjugated secondary antibodies against mouse, rabbit or rat immunoglobulin (GE Healthcare, Little Chalfont, Amersham, UK).

### Immunoprecipitation

For TNFRI complex I and necrosome IP, DISC buffer composed of 50 mM Tris-HCl (pH 7.5), 150 mM NaCl, 1 mM EDTA, 1% Triton X-100, protease inhibitors and 10% glycerol were used to lyse pretreated L929 or HT-29 cells. After the lysis step, each complex was purified by IP using 1 *μ*g of *α*-mTNFR1 (R&D systems, AF-425-PB) for TNFRI complex I, 1 *μ*g of *α*-mFADD (Santa cruz, sc-6036) for mouse necrosome or 1 *μ*g of *α*-hRIPK3 (ThermoFisher Scientific Pierce, PA1-41533) at 4 °C. In order to pull down each antibody-bound complex, protein G agarose beads (GE Healthcare, 17-0618-01) were incubated with cell lysates for 2 h, followed by 3x washing with DISC buffer. The immunoprecipitated complexes were eluted in boiled condition and analyzed by immunoblotting (antibodies used as indicated). Pull down of biotinylated Sorafenib was performed by adding DMSO, biotin or biotinylated Sorafenib (10/20/50/100 *μ*M) to L929sAhFas cell lysates or MEF cell lysates and incubating with Streptavidin Sepharose High Performance (GE Healthcare, 17-5113-01). Beads were blocked with 0.5% BSA (150 mM NaCl, 10 mM Tris-HCl pH 8, 10% glycerol, 0.5% BSA) before use. After incubation with cell lysates, streptavidin beads were washed with ice-cold NP-40 lysis buffer (150 mM NaCl, 10 mM Tris-HCl pH 8, 10% glycerol, 1% NP-40) and eluted by directly adding 2x Laemli buffer. Samples were boiled and analyzed by immunoblotting (antibodies used as indicated).

### Cell death analysis

Cell death was analyzed using a BD Pathway 855 high-content screening instrument (BD Biosciences) (first screening, Figure 1a).^73^ Ten thousand cells were seeded in a black-clear 96-well plate. The next day, the cells were pretreated with the indicated compounds for 1 h and then stimulated with mTNF, hTNF or agonistic anti-Fas Ab (concentration as indicated) in the presence of 3 *μ*M propidium iodide (Sigma-Aldrich) and 1 *μ*M Hoechst (Sigma-Aldrich). Images of at least 1000 cells were taken using a 10x objective and acquired data were analyzed using the Columbus software package. The percentage PI-positive nuclei was determined as a measure for cell death. For the second screening shown in Figure 1b, cell death was measured by incubating dying cells with Cell Titer Glo reagent (Promega, G7571) for 20 min, followed by analysis using a luminometer according to the manufacturer’s protocol. All data on L929sAhFas and MEF cells were analyzed using the BD Pathway imager.

### Systemic inflammatory response syndrome

All *in vivo* experiments were conducted according to institutional, national and European regulations. Animal protocols were approved by the ethics committee of Ghent University (SIRS model). Female C57BL/6 J mice (7–8 weeks old) were purchased from Janvier (Le Genest, France) for all SIRS experiments. To study the effect of Sorafenib on TNF-induced SIRS in mice, mice were challenged with 10 *μ*g mTNF (500 mg/kg) in the presence or absence of Sorafenib. Nec-1s served as a positive control, since it was shown to protect against TNF-induced SIRS.^42, 74^ Nec-1s (125 *μ*g; 6.25 mg/kg) was injected intravenously (i.v.) 15 min before mTNF-*α* challenge, whereas Sorafenib (10, 50 or 100 mg/kg) or vehicle was given by gavage 1,5 h before mTNF-*α* challenge. mTNF-*α* (10 *μ*g) was injected intravenously (i.v.). Nec-1s (100 mM stock in DMSO) and mTNF-*α* were diluted in endotoxin-free PBS and injected in a volume of 200 *μ*l, while Sorafenib was dissolved in an aqueous solution containing 8.75% ethanol and 12.5% Chremophor EL (as described earlier in Sonntag *et al.*^75^) and administrated in a total volume of 200 *μ*l. Rectal body temperature was recorded with an electric thermometer. Plasma samples were collected and cytokine (mTNF-*α* and mIL-6) concentrations were determined using a ProcartaPlex Multiplex Immunoassay (affymetrix, eBioscience) for Luminex200, according to manufacturer’s protocol. Two samples reached the maximum detection limit of Luminex and were given 30 000 pg/ml as concentration.

### Induction of renal ischemia–reperfusion injury

All *in vivo* experiments were conducted according to the protocols approved by the Protection of Animals Act (kidney IRI model). For all IRI experiments, 8-week-old male C57BL/6N mice (Charles River, Sulzfeld, Germany) were used 15 min before the onset of ischemia, mice received Sorafenib or vehicle in given concentrations in a total volume of 200 *μl* via intraperitoneal injection. Following inhalative induction narcosis with isoflurane, kidney IRI was performed as described previously.^44^ See also Supplementary Materials and Methods.

### Statistics

Two or three independent experiments were performed (as indicated in figure legends) and data were analyzed using GraphPad Prism 6. Values represent the mean values±standard error of the mean, unless indicated otherwise. For survival curve analysis, a Log-rank (Mantel-Cox) test was performed. One-way ANOVA and a Bonferroni multiple comparison test was performed where indicated. Statistical significance was accepted at *P*<0.05.

FACS, quantitative PCR, clonogenic assay, synthesis biotinylated Sorafenib. See Supplementary Material and Methods.
