# Supplementary material for: Sorafenib tosylate inhibits directly necrosome complex formation and protects in mouse models of inflammation and tissue injury
Source: Cell Death Dis. 2017 Jun 29;8(6):e2904–. doi: 10.1038/cddis.2017.298 (PMC5520944; doi:10.1038/cddis.2017.298)
Supplement: Supplementary Figures Legends [file cddis2017298x1.doc]

**Legends supplementary figures**

**Supplementary Figure S1. Schemes of workflow screening assays in L929 cell lines.** (A) Scheme of the workflow of a high-content screening assay on L929sAhFas cells using mTNF (T) and mTNF+Tak1i ((5Z)-7-Oxozeaenol) (TiT) to induce necroptotic cell death. Nec-1 and zVAD-fmk were used as protective and sensitizing benchmarks, respectively, on all plates. (B) Scheme of the workflow of a CellTiter-Glo luminescent cell viability screening assay on L929 cells using mTNF+zVAD (zVT) to induce necroptotic cell death.

**Supplementary Figure S2. Z’ calculation for cell death screening assays in L929 cells.** (A) Z’ calculation for cell death screening assay (Fig. 1A) in mTNF-stimulated L929sAhFas cells. (B) Z’ calculation for cell death screening assay (Fig. 1A) in L929sAhFas cells stimulated with mTNF + Taki.

Supplementary Figure S3. Sorafenib is cytotoxic at concentrations higher than 25 µM. L929sAhFas cells were treated with DMSO, Nec-1s or Sorafenib (as indicated) for 19 h, 24 h or 48 h. Data represent mean values ± S.E.M. of two independent experiments. Cells were stained with Hoechst and PI and percentage PI-positive nuclei was determined using high-content image analysis (BD pathway Bioimager).

**Supplementary Figure S4. Sorafenib inhibits TNF-induced RIPK1-dependent cell death in both murine and human cell lines and rescues AML cells from Smac mimetic-induced necroptosis.** L929sAhFas (A) and MEF (B) cells were pretreated for 1 h with DMSO, Nec-1s or Sorafenib (concentration as indicated) and stimulated for 4.25 h (L929) or 3 h (MEF) with 38 ng/mL mTNF, 500 ng/mL anti-Fas agonistic Ab (AF) and 1 µM BV6 as indicated. Data were normalized to DMSO-treated control cells and represent the mean value ± S.E.M. of three independent experiments. Toxic concentrations were removed from the analysis. Cells were stained with Hoechst and PI and percentage PI-positive nuclei was determined using high-content image analysis (BD pathway Bioimager). (C-D) Molm13 and MV4-11 cells were treated with Sorafenib (concentration as indicated) as single agent. Cell death was determined by PI staining and flow cytometry. Mean and SD of three independent experiments performed in triplicate are shown.

**Supplementary Figure S5. Sorafenib does not influence the TNF-induced NF-κB pathway.** L929sAhFas cells were pretreated with 10 µM Nec-1s, Sorafenib or DMSO for 1 h and stimulated with mTNF (3.8 ng/mL) for the time indicated. Cells were lysed and immunoblotted with the indicated antibodies. r.p.a. = relative protein amount (int. IκB-α/int. actin normalized to timepoint 0). * = phosphorylated RIPK1.

**Supplementary Figure S6. Sorafenib does not influence A20 and IκB-α gene expression, but does reduce mTNF α, mMIP-2, mMCP1 and mCXCL1 gene expression after mTNF stimulation.** L929sAhFas cells were pretreated with 10 µM DMSO, Nec-1 or Sorafenib for 1 h and stimulated with mTNF (0.5 ng/mL) for 2 h. The relative mRNA levels of mA20, mIκB-α, mTNFα, mMIP-2, mMCP1 and mCXCL1 were analyzed by qRT-PCR. All bars represent mean ± S.E.M.; n = 3, *** p< 0.001 ****p<0.0001. Statistical analysis according to Willems et al., 2008, Anal. Biochem. .

**Supplementary figure S7. Sorafenib protects against necroptosis by targeting the necrosome complex.** L929sAhFas cells were pretreated with 10 µM Nec-1s, Sorafenib or DMSO and stimulated with FLAG-hTNF (1.5 µg/mL) for 5 minutes. Cell lysates were subjected to immunoprecipitation with α-FLAG-beads and immunoprecipitates (IP) and total lysate were immunoblotted with the indicated antibodies (IB).

**Supplementary Figure S8. Synthesis of Sorafenib analogues and their ability to inhibit necroptosis.** (A) General scheme: synthesis of Sorafenib analogues (6-9) and biotinylated derivative (10). (B) Table: anti-necroptotic activity of synthesized Sorafenib analogues. L929sAhFas cells were pre-treated with every Sorafenib analogue for 1 h or 24 h (-1h/-24h) and stimulated with 1000 IU/ml mTNF for 4 h. IC50 values were determined using nonlinear regression curve fitting in GraphPad Prism 6.

**Supplementary Figure S9. Quantification of pull-down of biotinylated Sorafenib in L929sAhFas cells.** Quantification of bands detected after streptavidin pull-down with biotinylated Sorafenib in L929sAhFas cells. Relative band intensities were calculated using ImageJ. For each protein, the intensity of the bands was expressed relative to the intensity of the band at 10 µM biotinylated Sorafenib.

**Supplementary Figure S10. Sorafenib does not protect against ligand-independent MLKL-induced cell death in L929sAhFas cells.** L929sAhFas MLKL-/- with reconstituted inducible WT MLKL-Flag (A-B) or MLKL-S345D-Flag (C-D) were stimulated with doxycyclin (1 µg/mL) (DOX) for 5 hours and then treated with DMSO/Sorafenib (10 µM). Cell death was measured over time using SytoxGreen staining. Measurement started from the moment compound was added (5 hours after doxycyclin stimulation). Percentage of cell death is expressed as percent of control (POC) of maximal SytoxGreen fluorescence (Tx100). Cell lysates were collected at the indicated timepoints and immunoblotted for indicated antibodies. Representative results of 2 independent experiments.

**Supplementary Figure S11. High dose Sorafenib sensitizes to ischemic damage and deterioration of acute renal failure.** Serum urea/creatinine levels of mice treated with vehicle or Sorafenib (100 mg/kg i.p.) 15 min before the initiation of ischemia.
