# Supplementary material for: Sorafenib tosylate inhibits directly necrosome complex formation and protects in mouse models of inflammation and tissue injury
Source: Cell Death Dis. 2017 Jun 29;8(6):e2904–. doi: 10.1038/cddis.2017.298 (PMC5520944; doi:10.1038/cddis.2017.298)
Supplement: Supplementary Material and Methods [file cddis2017298x2.doc]

**Supplementary materials and methods**

**Immunoblotting.** After treatments, cells were washed twice with PBS and lysed with Laemmli buffer (2x) directly or with NP-40 lysis buffer (150 mM NaCl, 1% NP-40, 10% glycerol, 10 mM Tris-HCl pH 8) supplemented with EDTA-free protease inhibitor cocktail tablets (Roche Diagnostics, Basel, Switzerland, #11873580001) and phosphatase inhibitor cocktail tablets (Roche Diagnostics, #04906837001). After lysis with NP-40 lysis buffer, lysates were kept 10 minutes on ice and centrifugated in a microcentrifuge (10 min, 13.000 rpm, 4°C). Supernatant was used for SDS-PAGE and immunoblotting.

**FACS analysis.** FACS analysis was used to measure dying HT-29, Jurkat FADD-/- and AML cells. Treated HT-29 cells were harvested, washed with PBS and incubated with Annexin V binding buffer (BD Biosciences, 556454) containing 7-AAD and Annexin V-FITC for 15 min according to the manufacturer’s protocol. FACSCalibur^TM^ Flow Cytometer (BD Biosciences) and CellQuest Pro software were used to perform experiments and analyze data respectively.

**Time kinetic cell death analysis.** For time kinetics, cell death was analyzed on a FLUOSTAR Omega (BMG Labtech, Offenburg, Germany). Using this technique, cells were stained with 5 µM SytoxGreen (Life Technologies) and 33 µM ac-DEVD-amc (PeptaNova GmbH, Sandhausen, Germany) after treatment with doxycyclin and compounds as indicated. Maximal cell death was obtained by treatment with Triton-X100 (0.05%). Cell death and caspase activity was determined as described previously (1).

**Clonogenic assay.** L929sAhFas cells were treated with Sorafenib (concentration as indicated) for 24h. Next, treatment was removed and 50 cells/condition were seeded. After 10 days, cells were fixed with 4% PFA for 10 minutes. After fixation, cells were stained with a 0.01% crystal violet solution for 1 hour. Cells were washed with PBS and images were taken. Colonies were counted using ImageJ.

**Quantitative PCR.** Trizol (Invitrogen, BRL-15596-018) was used for the extraction of RNA from L929 cells according to the manufacturer’s protocol. cDNA of L929 cells was synthesized from 1 µg of total RNA using PrimeScript^TM^ Reverse Transcriptase (Takara Bio, 2680A). The synthesized cDNA was analyzed with QuantiTect SYBR Green PCR Kit (Qiagen, 204143) according to the manufacturer’s guidelines. mRNA levels of mA20 and mIκB-α were normalized to mGAPDH.

Rneasy mini-kit (Qiagen, 74106) was used for the extraction of RNA from L929sAhFas cells according to the manufacturer’s protocol. cDNA of L929sAhFas cells was synthesized from 1 µg of total RNA using the Sensifast cDNA synthesis kit (Bioline, BIO-650504), Dnase digestion (RNase-Free DNase Set, Qiagen, 79254) on-column was included. The synthesized cDNA was analyzed with SensiFast^TM^ SYBR No-Rox Kit (GC Biotech, CSA-01190) according to the manufacturer’s guidelines. mRNA levels of mA20, mIκB-α, mTNF-a, mMCP1, mMIP-2, mCXCL-1 were normalized to multiple reference genes (mTBP, mRPL13a, mB2M, mHMBS, mACTB) using qbase+ software. Statistical analysis according to *Willems et al., 2008, Anal. Biochem.*.

**Induction of renal ischemia-reperfusion injury (IRI).** For all IRI experiments, 8-week-old male C57BL/6N mice (Charles River, Sulzfeld, Germany) were used. 15 min. prior to the onset of ischemia, mice received Sorafenib or vehicle in given concentrations in a total volume of 200µL via intraperitoneal injection. Following inhalative induction narcosis with isoflurane, kidney IRI was performed as described previously (2). Briefly, we performed a midline abdominal incision and a bilateral renal pedicle clipping for 30 min using microaneurysm clamps (Aesculab, Tuttlingen, Germany). Throughout the surgical procedure, the body temperature was maintained between 37.0 and 38.5 degrees Celsius by continuous monitoring using a temperature-controlled self-regulated heating system (Fine Science Tools, Heidelberg, Germany). After removal of the clamps, reperfusion of the kidneys was visually confirmed. The abdomen was closed in two layers using standard 6-0 sutures. Sham-operated mice received identical surgical procedures, except that microaneurysm clamps were not applied. To maintain fluid balance, all of the mice were supplemented with 1 ml of prewarmed phosphate-buffered saline administered intraperitoneally directly after surgery. The mice were killed 48 h after reperfusion if not otherwise specified for each experiment.

Kidneys were removed after retroorbital blood puncture and were fixed with 4% neutral-buffered formaldehyde, for 24 h, dehydrated in a graded ethanol series and xylene, and finally embedded in paraffin. Paraffin sections (3–5 mm) were stained with periodic acid–Schiff reagent, according to the standard routine protocol. Stained sections were analyzed using an Axio Imager microscope (Zeiss, Oberkochen, Germany) at 200 fold and 400 fold original magnification. Micrographs were digitalized using an AxioCam MRm Rev. 3 FireWire camera and AxioVision Rel. 4.5 software (Zeiss). Organ damage was quantified by an experienced pathologist in a double-blind manner on a scale ranging from 0 (unaffected tissue) to 10 (severe organ damage) as described previously (3).

**Synthesis of biotinylated Sorafenib (Suppl. Figure S8)**

The synthesis of biotinylated Sorafenib and its analogues is outlined in the general scheme (Suppl. Figure S5). Starting from the commercially available 4-chloropicolinic acid (**1),** a *tert*-butyl protecting group was introduced to the carboxylic acid moiety of **(1),** resulting in intermediate (**2)**. Next, *para*-aminophenol was coupled to (**2)** in basic conditions, resulting in compound (**3).** Intermediate (**4)** was obtained by reaction of (**3)** with 4-chloro-3-(trifluoromethyl)phenylisocyanate. Removing the *tert*-butyl protecting group under acid conditions yielded carboxylic acid intermediate (**5)**. Various primary amines were coupled to the carboxylic acid using HATU, resulting in target compounds (**6-10)**.

The following section describes the synthetic procedures and analytical data for all compounds reported in this manuscript. Synthesis procedures used in the preparation of multiple intermediates and final products are summarized as “General Procedures.”

**Tert-butyl 4-chloropicolinate (2)**

4-chloropicolinic acid (**1)** (66.6 mmol) was suspended in thionyl chloride (566 mmol). Dimethylformamide (0.256 ml, 3.33 mmol) was added dropwise. The mixture was refluxed for 2 h. Excess thionyl chloride was removed under reduced pressure to give the pale yellow acyl chloride. The resulting crude was dissolved in dichloromethane (6 ml) and added to mixture of *tert*-butanol (2.5 ml), pyridine (2 ml) and dichloromethane (8 ml) at ‒40°C. Afterwards, the reaction mixture was heated to 50°C and stirred for 16 h. The solvents were removed under reduced pressure and ethyl acetate was added. The resulting mixture was washed with saturated brine, sodium hydroxide solution (1N). The organic phase was dried over anhydrous sodium sulfate and concentrated under reduced pressure. The residue was dried under vacuum to give tert-butyl 4-chloropicolinate (70.2 % yield) as a dark brown solid.

**^1^H NMR (400 MHz, DMSO*-d_6_*)** δ 1.56 (s, 9H), 7.78 (dd, J = 2.10, 5.25 Hz, 1H), 8.00 (dd, J = 0.61, 2.08 Hz, 1H), 8.68 (dd, J = 0.62, 5.25 Hz, 1H) **^13^C NMR (101 MHz, DMSO*-d_6_*)** δ 28.09, 82.57, 124.94, 127.38, 144.41, 150.90, 151.52, 163.11. t_R_ 2.30 min, MS (ESI) m/z 214 [M +H]

**Tert-butyl 4-(4-aminophenoxy)picolinate (3)**

*Para*-aminophenol (14.04 mmol) was dissolved in DMF (30 ml) at room temperature. To the resulting mixture, *tert*-butylalcohol,potassiumderivative (14.04 mmol) was added gradually and the mixture was stirred for 0.5 h. *Tert*-butyl 4-chloropicolinate (**2)** (14.04 mmol) and K_2_CO_3_ (1.00 mmol) were added. The reaction mixture was heated to 80°C and stirred for 1 h. The mixture was cooled down to room temperature and ethyl acetate was added. The mixture was filtered to remove the undissolved material and the filtrate was washed with brine. The organic phase was dried over anhydrous sodium sulfate and concentrated under reduced pressure. The residue was purified by column chromatography to give *tert-*butyl 4-(4-aminophenoxy)picolinate (99 % yield).

**^1^H NMR (400 MHz, DMSO*-d_6_*)** δ 1.52 (s, 9H), 5.18 (s, 2H), 6.70 - 6.60 (m, 2H), 6.93 - 6.82 (m, 2H), 7.04 (dd, J = 2.57, 5.59 Hz, 1H), 7.36 (dd, J = 0.51, 2.55 Hz, 1H), 8.50 (dd, J = 0.51, 5.61 Hz, 1H) **^13^C NMR (101 MHz, DMSO*-d_6_*)** δ 28.13, 82.00, 112.07, 114.44, 115.33, 121.96, 143.25, 147.36, 151.26, 151.67, 164.13, 166.77. t_R_ 1.73 min, MS (ESI) m/z 287 [M +H].

**Tert-butyl 4-(4-(3-(4-chloro-3-(trifluoromethyl)phenyl)ureido)phenoxy)picolinate (4)**

4-chloro-3-(trifluoromethyl)phenylisocyanate (7.81 mmol) was dissolved in DCM at room temperature. The resulting solution was combined with a solution of *tert*-butyl 4-(4-aminophenoxy)picolinate (**3)** (7.44 mmol) in DCM. The mixture was stirred for 16 h at room temperature. The solvent was removed under reduced pressure, and the resulting solid was purified by column chromatography to give *tert*-butyl 4-(4-(3-(4-chloro-3-(trifluoromethyl)phenyl)ureido)phenoxy)picolinate (96 % yield).

**^1^H NMR (400 MHz, DMSO*-d_6_*)** δ 1.53 (s, 9H), 7.13 (dd, J = 2.56, 5.61 Hz, 1H), 7.18 (dd, 2H), 7.42 (dd, J = 0.51, 2.58 Hz, 1H), 7.60 (dd, J = 2.57, 9.32 Hz, 2H), 7.67 - 7.63 (m, 2H), 8.13 (d, J = 2.46 Hz, 1H), 8.55 (dd, J = 0.50, 5.62 Hz, 1H), 9.06 (s, 1H), 9.28 (s, 1H) **^13^C NMR (101 MHz, DMSO*-d_6_*)** δ 28.13, 82.12, 112.48, 114.83, 120.95, 121.82, 122.83, 123.59, 132.47, 137.52, 139.79, 148.28, 151.42, 151.89, 152.93, 164.02, 165.93. t_R_ 2.23 min, MS (ESI) m/z 508 [M +H].

**4-(4-(3-(4-chloro-3-(trifluoromethyl)phenyl)ureido)phenoxy)picolinic acid (5)**

*Tert*-butyl 4-(4-(3-(4-chloro-3-(trifluoromethyl)phenyl)ureido)phenoxy)picolinate **4** (7.29 mmol) was dissolved in DCM (53 ml) at room temperature. To the resulting solution trifluoroaceticacid (7.29 mmol) and triethylsilane (7.29 mmol) were added. The resulting mixture was heated to 50°C and stirred for 16 h. The solvent was removed under reduced pressure and water and ethyl acetate were added. The resulting mixture was separated and the organic phase was removed. The aqueous layer was filtered and the solid was washed with water. The solid was dried to give 4-(4-(3-(4-chloro-3-(trifluoromethyl)phenyl)ureido)phenoxy)picolinic acid (60.8 % yield).

**^1^H NMR (400 MHz, DMSO*-d_6_*)** δ 7.26 - 7.15 (m, 3H), 7.47 (dd, J = 0.50, 2.58 Hz, 1H), 7.65 - 7.58 (m, 4H), 8.14 (d, J = 2.52 Hz, 1H), 8.59 (dd, J = 0.53, 5.70 Hz, 1H), 9.18 (s, 1H), 9.39 (s, 1H) **^13^C NMR (101 MHz, DMSO*-d_6_*)** δ 112.03, 114.75, 116.77, 116.83, 120.51, 120.98, 121.41, 122.35, 123.06, 131.98, 137.36, 139.40, 147.58, 149.86, 150.30, 152.51, 165.02, 166.46. t_R_ 1.73 min, MS (ESI) m/z 452 [M +H].

**HATU coupling of 4-(4-(3-(4-chloro-3-(trifluoromethyl)phenyl)ureido)phenoxy)picolinic acid with different amine analogues. General Procedure:** 4-(4-(3-(4-chloro-3-(trifluoromethyl)phenyl)ureido)phenoxy)picolinic acid **5** (0.443 mmol) was dissolved in DMF at room temperature. The resulting solution was combined with the desired primary amine (0.885 mmol), HATU (0.855 mmol) and *N*,*N*-Di-iso-propylethylamine (2.66 mmol). The resulting mixture was stirred at room temperature for 16 h. After reaction, water was added and the resulting mixture was stirred for 0.5 h. The mixture was then filtered to give a white solid. The solid was dissolved in ethyl acetate, and the resulting mixture was washed with brine and then separated. The organic phase was dried over anhydrous sodium sulfate, concentrated under reduced pressure. The resulting crude was purified by silica column chromatography to give the desired Sorafenib analogues (**6-10)**.

**4-(4-(3-(4-chloro-3-(trifluoromethyl)phenyl)ureido)phenoxy)-*N*-methylpicolinamide (6)**

General procedure using methylamine hydrochloride; yield: 50.1%.

**^1^H NMR (400 MHz, DMSO*-d_6_*)** δ 2.79 (d, J = 4.81 Hz, 3H), 7.22 - 7.12 (m, 3H), 7.39 (d, J = 2.57 Hz, 1H), 7.69 - 7.57 (m, 4H), 8.13 (d, J = 2.43 Hz, 1H), 8.51 (d, J = 5.60 Hz, 1H), 8.77 (d, J = 5.33 Hz, 1H), 9.01 (s, 1H), 9.22 (s, 1H) **^13^C NMR (101 MHz, DMSO*-d_6_*)** δ 26.48, 99.99, 109.14, 114.50, 117.33, 120.99, 121.94, 122.83, 123.60, 127.03, 127.33, 132.48, 137.52, 139.80, 148.30, 150.85, 152.95, 164.26, 166.43. t_R_ 1.94 min, MS (ESI) m/z 452 [M +H]. (95%)

**4-(4-(3-(4-chloro-3-(trifluoromethyl)phenyl)ureido)phenoxy)-*N*-ethylpicolinamide (7)**

General procedure using ethylamine hydrochloride; yield: 35.9%.

**^1^H NMR (400 MHz, DMSO*-d_6_*)** δ 1.14 - 1.06 (m, 3H), 3.32 - 3.25 (m, 2H), 7.21 - 7.13 (m, 3H), 7.39 (dd, J = 0.55, 2.58 Hz, 1H), 7.70 - 7.57 (m, 4H), 8.13 (d, J = 2.42 Hz, 1H), 8.51 (dd, J = 0.55, 5.55 Hz, 1H), 8.81 (t, J = 6.06 Hz, 1H), 9.05 (s, 1H), 9.27 (s, 1H) **^13^C NMR (101 MHz, DMSO*-d_6_*)** δ 15.28, 34.18, 109.17, 114.56, 117.31, 120.98, 121.93, 122.80, 123.59, 127.02, 127.33, 132.48, 137.55, 139.83, 148.30, 150.80, 152.96, 153.00, 163.45, 166.46. t_R_ 2.02 min, MS (ESI) m/z 479 [M +H]. (100%)

**4-(4-(3-(4-chloro-3-(trifluoromethyl)phenyl)ureido)phenoxy)-*N*-butylpicolinamide (8)**

General procedure using butylamine hydrochloride; yield 46.3%.

**^1^H NMR (400 MHz, CDCl_3_)** δ 0.92 (t, J = 7.35 Hz, 3H), 1.33 - 1.46 (m, 2H), 1.58 - 1.69 (m, 2H), 3.47 (td, J = 5.91, 7.09 Hz, 2H), 6.91 - 6.97 (m, 2H), 7.09 (dd, J = 2.49, 5.59 Hz, 1H), 7.29 - 7.36 (m, 3H), 7.56 - 7.62 (m, 2H), 7.67 (d, J = 2.54 Hz, 1H), 8.31 (s, 1H), 8.41 (t, J = 6.05 Hz, 1H), 8.45 (dd, J = 0.49, 5.48 Hz, 1H), 8.57 (s, 1H) **^13^C NMR (101 MHz, CDCl_3_)** δ 13.63, 20.11, 31.41, 39.70, 108.49, 115.21, 117.98, 120.95, 121.35, 123.03, 125.00, 128.30, 128.61, 131.79, 136.69, 138.05, 148.31, 150.13, 151.28, 153.02, 164.88, 166.82. t_R_ 2.30 min, MS (ESI) m/z 507 [M +H]. (95%)

**4-(4-(3-(4-chloro-3-(trifluoromethyl)phenyl)ureido)phenoxy)-*N*-(2-(2-methoxyethoxy)ethyl)picolinamide (9)**

General procedure using 2-(2-methoxyethoxy)ethylamine; yield: 68.7%.

**^1^H NMR (400 MHz, DMSO*-d_6_*)** δ 3.24 (s, 3H), 3.40 - 3.48 (m, 4H), 3.49 - 3.56 (m, 4H), 7.14 - 7.22 (m, 3H), 7.40 (dd, J = 0.53, 2.68 Hz, 1H), 7.57 - 7.70 (m, 4H), 8.13 (d, J = 2.43 Hz, 1H), 8.52 (dd, J = 0.56, 5.60 Hz, 1H), 8.71 (t, J = 5.88 Hz, 1H), 9.01 (s, 1H), 9.23 (s, 1H) **^13^C NMR (101 MHz, DMSO*-d_6_*)** δ 58.53, 69.17, 69.79, 71.70, 109.22, 114.69, 117.33, 120.99, 121.93, 122.83, 123.59, 124.65, 127.03, 127.34, 132.47, 137.55, 139.80, 148.29, 150.88, 152.62, 152.94, 163.71, 166.50. t_R_ 2.06 min, MS (ESI) m/z 552 [M +H]. (100%)

**4-(4-(3-(4-chloro-3-(trifluoromethyl)phenyl)ureido)phenoxy)-*N*-(2-(2-(2-(5-(2-oxohexahydro-1*H*-thieno[3,4-d]imidazol-4-yl)pentanamido)ethoxy)ethoxy)ethyl)picolinamide (10)**

General procedure using biotinylated amine analogue; yield: 53.5%.

**^1^H NMR (400 MHz, DMSO*-d_6_*)** δ 1.25 - 1.31 (m, 2H), 1.43 - 1.66 (m, 4H), 2.06 (t, J = 7.38 Hz, 2H), 2.58 (d, J = 12.43 Hz, 1H), 2.81 (dd, J = 5.09, 12.42 Hz, 1H), 3.05 - 3.11 (m, 1H), 3.14 - 3.21 (m, 2H), 3.37 - 3.46 (m, 4H), 3.48 - 3.57 (m, 6H), 4.12 (ddd, J = 1.87, 4.42, 7.74 Hz, 1H), 4.27 - 4.33 (m, 1H), 6.32 - 6.45 (m, 2H), 7.14 - 7.22 (m, 3H), 7.40 (dd, J = 0.54, 2.57 Hz, 1H), 7.56 - 7.71 (m, 4H), 7.82 (t, J = 5.64 Hz, 1H), 8.13 (d, J = 2.40 Hz, 1H), 8.53 (dd, J = 0.55, 5.56 Hz, 1H), 8.72 (t, J = 5.89 Hz, 1H), 9.07 (s, 1H), 9.30 (s, 1H) **^13^C NMR (101 MHz, MeOD)** δ 25.47, 28.10, 28.37, 35.35, 38.99, 39.71, 42.40, 54.43, 55.62, 60.22, 61.96, 69.24, 69.93, 109.42, 113.90, 117.32, 120.93, 121.15, 121.56, 122.84, 123.98, 124.26, 127.73, 128.03, 131.60, 136.80, 138.85, 148.82, 150.20, 151.80, 153.27, 164.67, 164.77, 166.68, 171.58, 174.66. t_R_ 1.94 min, MS (ESI) m/z 809 [M +H]. (95%)

Chang Jun Yu. U.S. Application No. US 2009/0012091 A1. 2009.

**References**

1. Remijsen Q*, et al.* (2014) Depletion of RIPK3 or MLKL blocks TNF-driven necroptosis and switches towards a delayed RIPK1 kinase-dependent apoptosis. *Cell Death Dis* 5:e1004.

2. Linkermann A*, et al.* (2012) Rip1 (receptor-interacting protein kinase 1) mediates necroptosis and contributes to renal ischemia/reperfusion injury. *Kidney Int* 81(8):751-761.

3. Linkermann A*, et al.* (2013) Two independent pathways of regulated necrosis mediate ischemia-reperfusion injury. *Proc Natl Acad Sci U S A* 110(29):12024-12029.
