# Supplementary material for: Sorafenib tosylate inhibits directly necrosome complex formation and protects in mouse models of inflammation and tissue injury
Source: Cell Death Dis. 2017 Jun 29;8(6):e2904–. doi: 10.1038/cddis.2017.298 (PMC5520944; doi:10.1038/cddis.2017.298)
Supplement: Supplementary Figures and Tables [file cddis2017298x3.pdf]

Supplementary Figures

Supplementary Figure S1.

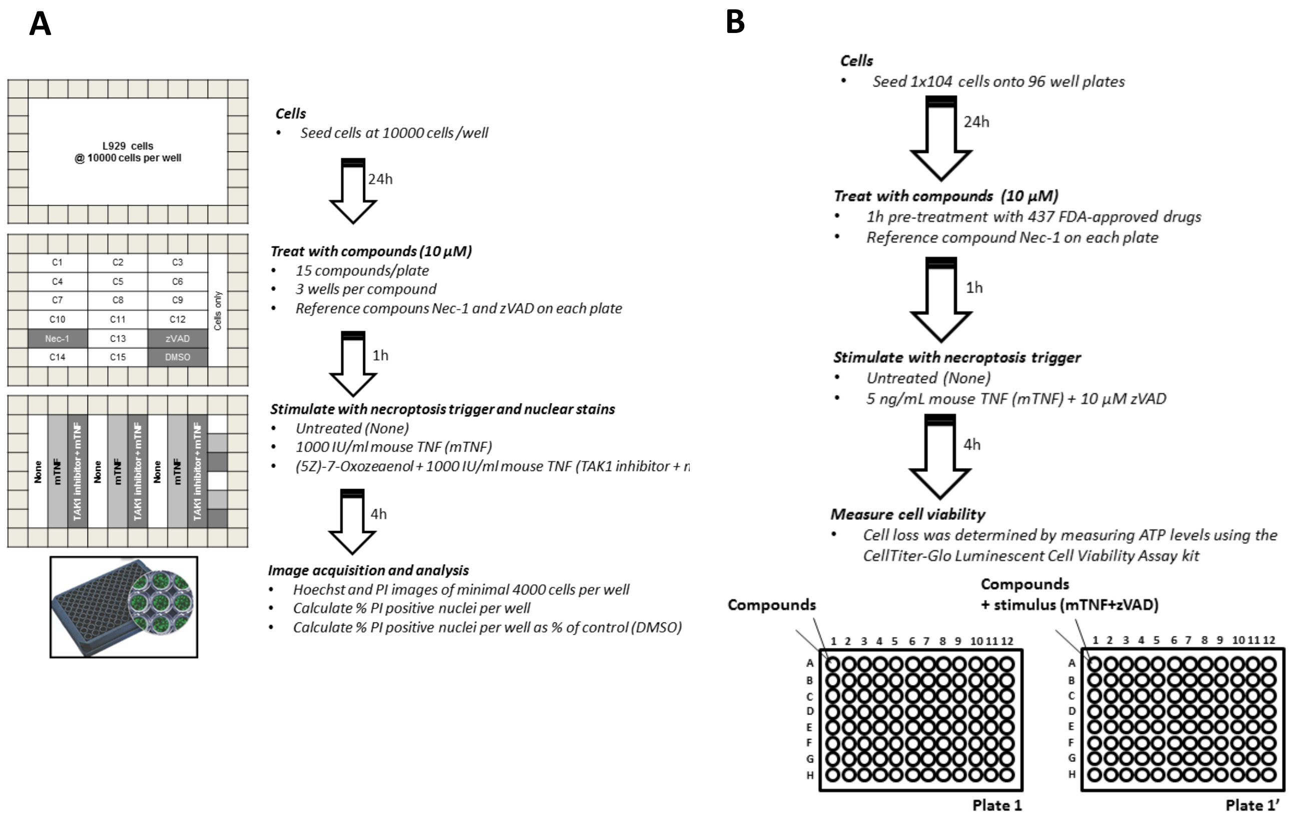

Supplementary Figure S2.

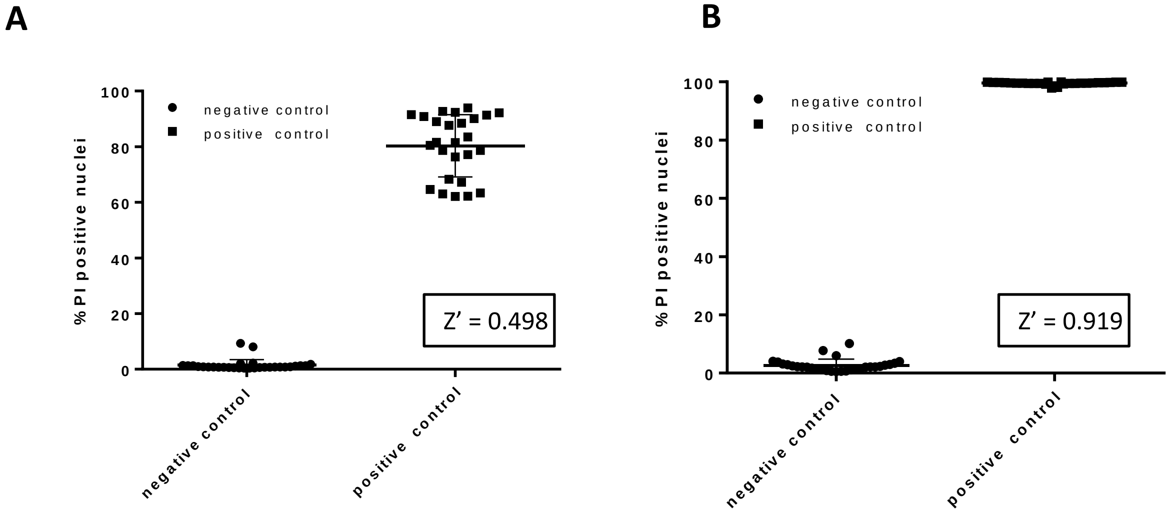

Supplementary Figures

Supplementary Figure S3.

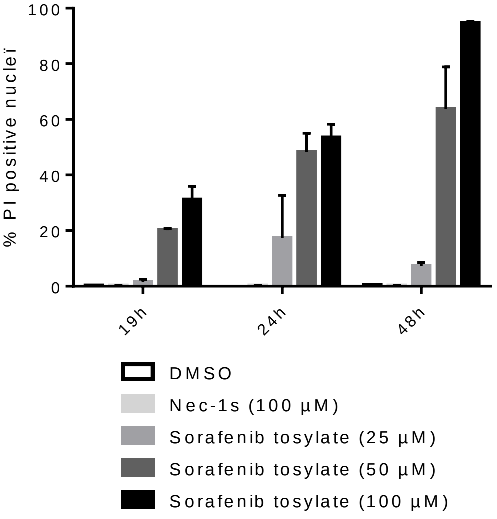

Supplementary Figure S4.

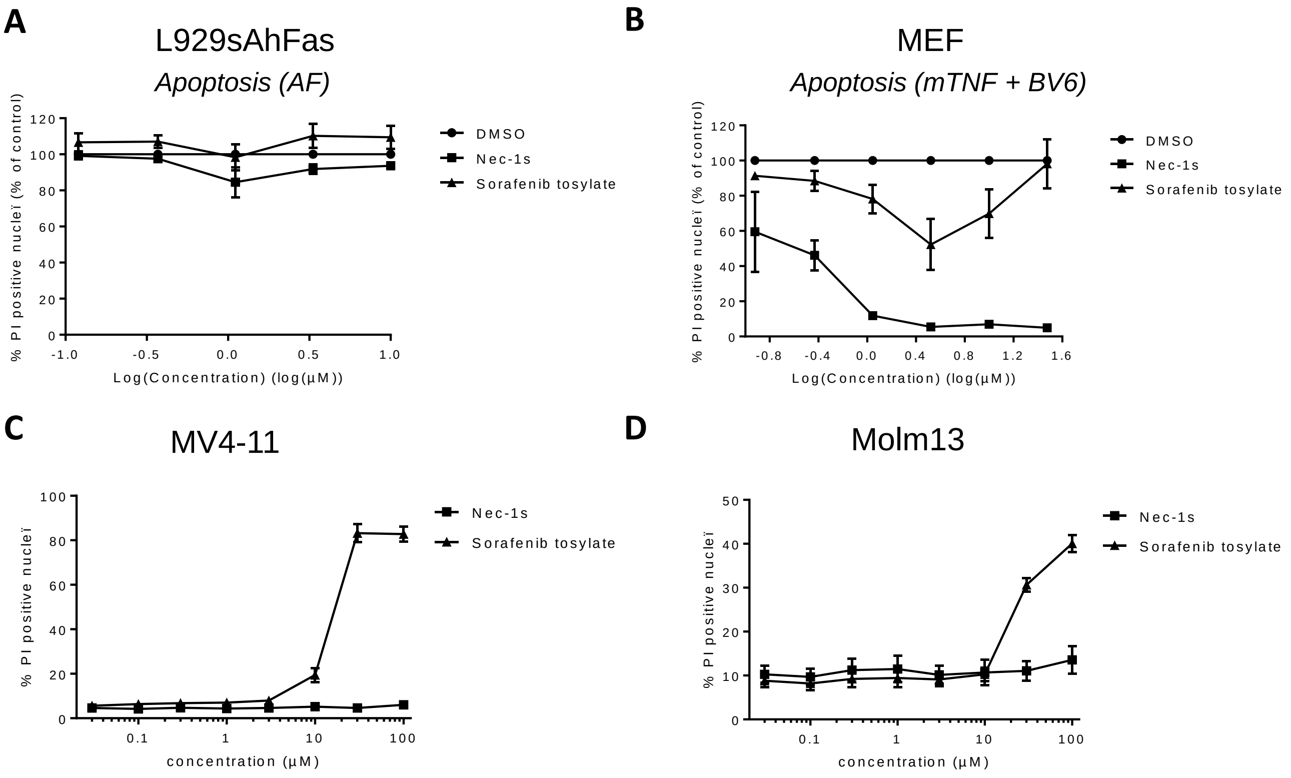

Supplementary Figures

Supplementary Figure S5.

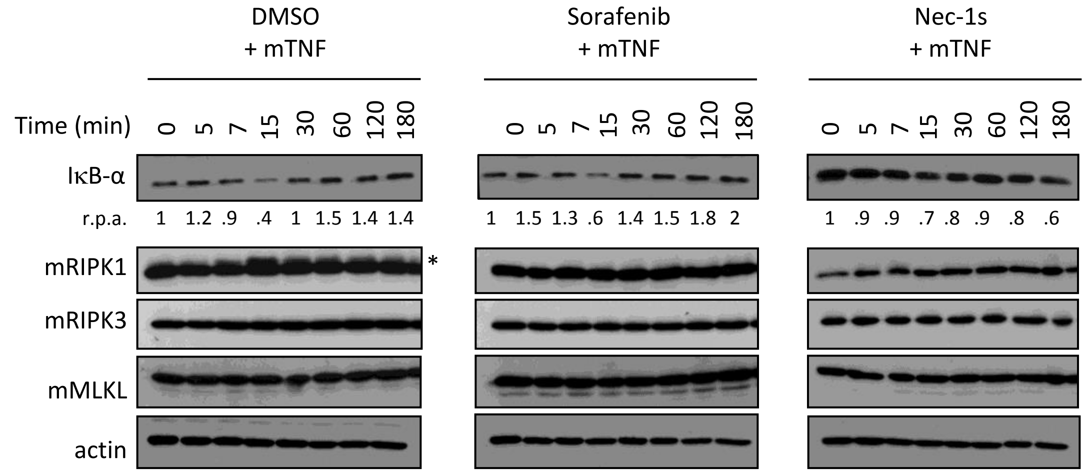

Supplementary Figure S6.

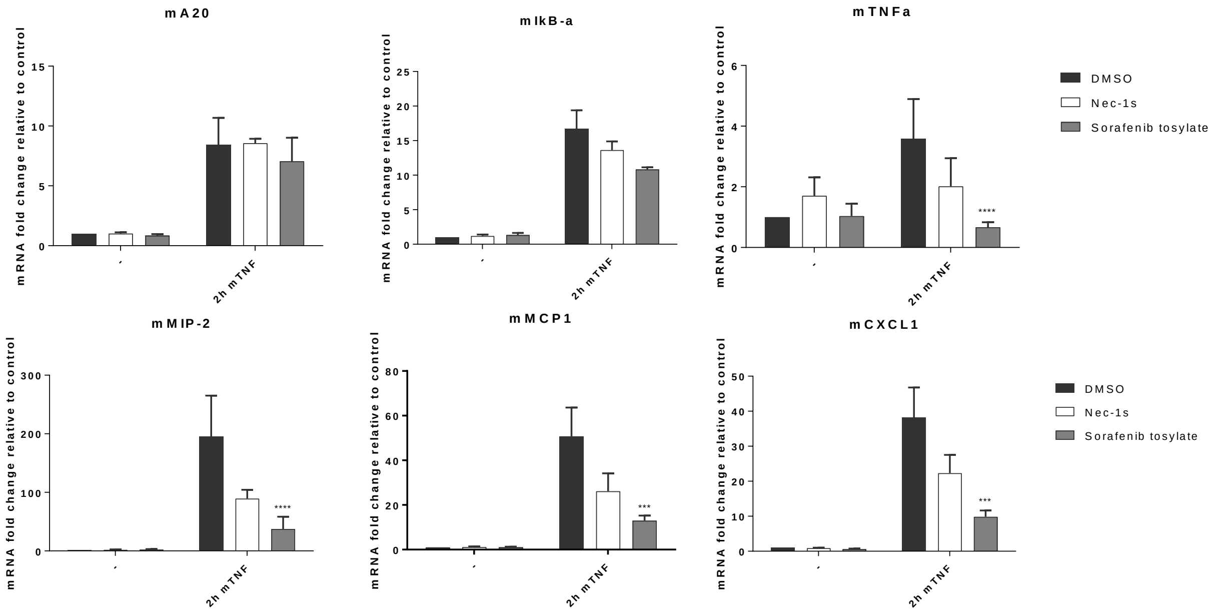

Supplementary figure S7.

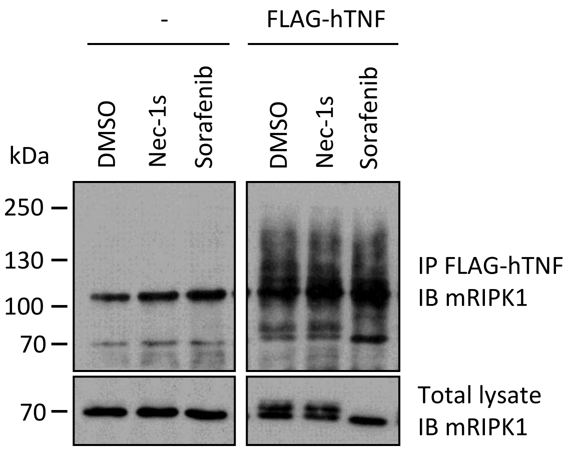

Supplementary Figures

Supplementary Figure S8.

A

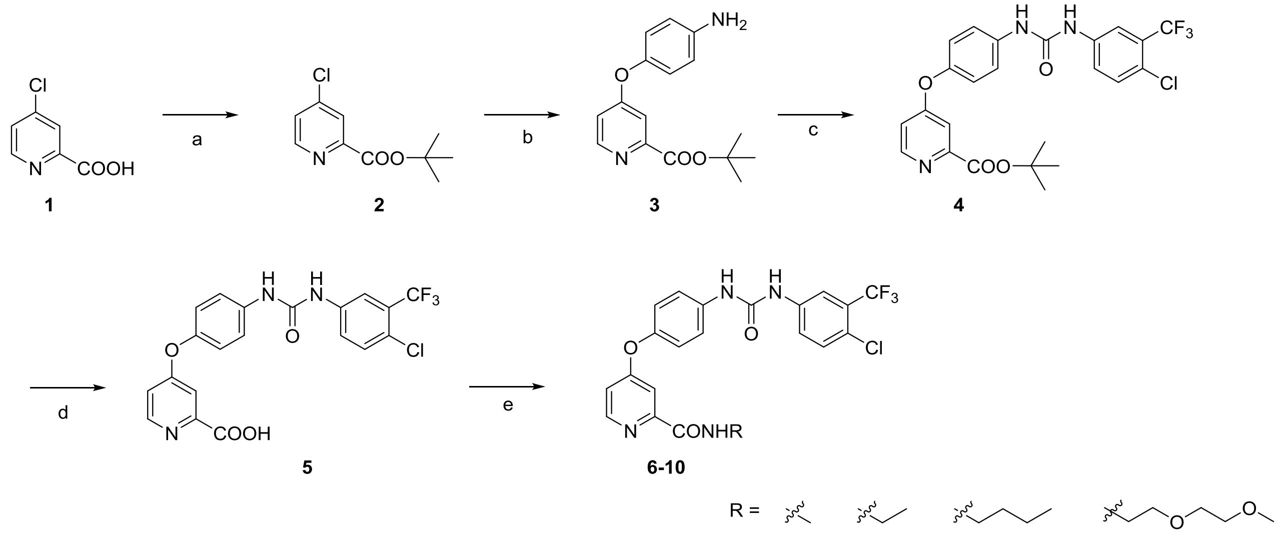

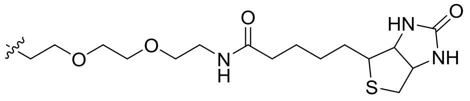

Reagents and conditions:

a: 1. $SOCl_2$, DMF, reflux, 2h;
  2. *tert.*-BuOH, pyridine, DCM, -40°C to 50°C, 16h;
b: 1. *p*-aminophenol, *tert.*-BuOK, DMF, rt, 0.5h;
  2. *tert.*-butyl 4-chloropicolinate, K_2CO_3, 80°C, 2h;
c: 4-chloro-3-(trifluoromethyl)phenylisocyanate, DCM, rt, 16h;
d: TFA, triethylsilane, DCM, 50°C, 16h
e: Primary amine analogue, HATU, DIPEA

B

Compound	R	IC_{50} (μM)	
		-1h	-24h
6	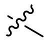	1.29	1.42
7	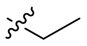	2.24	>10
8	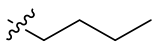	4.65	>10
9	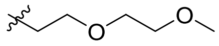	1.50	n/a
10	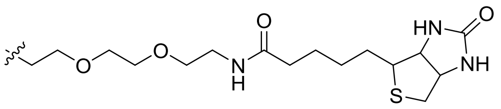	n/a	>10

Supplementary Figures

Supplementary Figure S9.

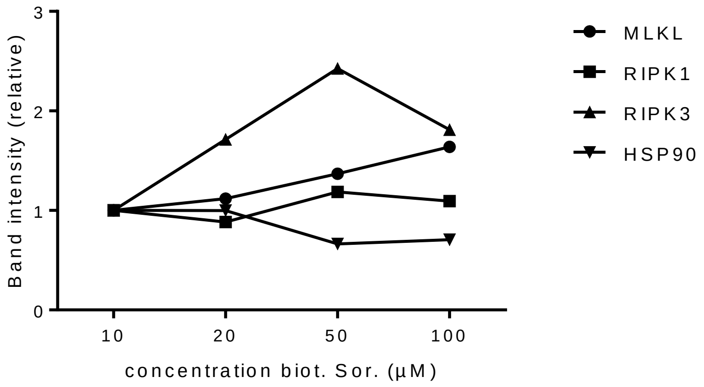

Supplementary Figure S10.

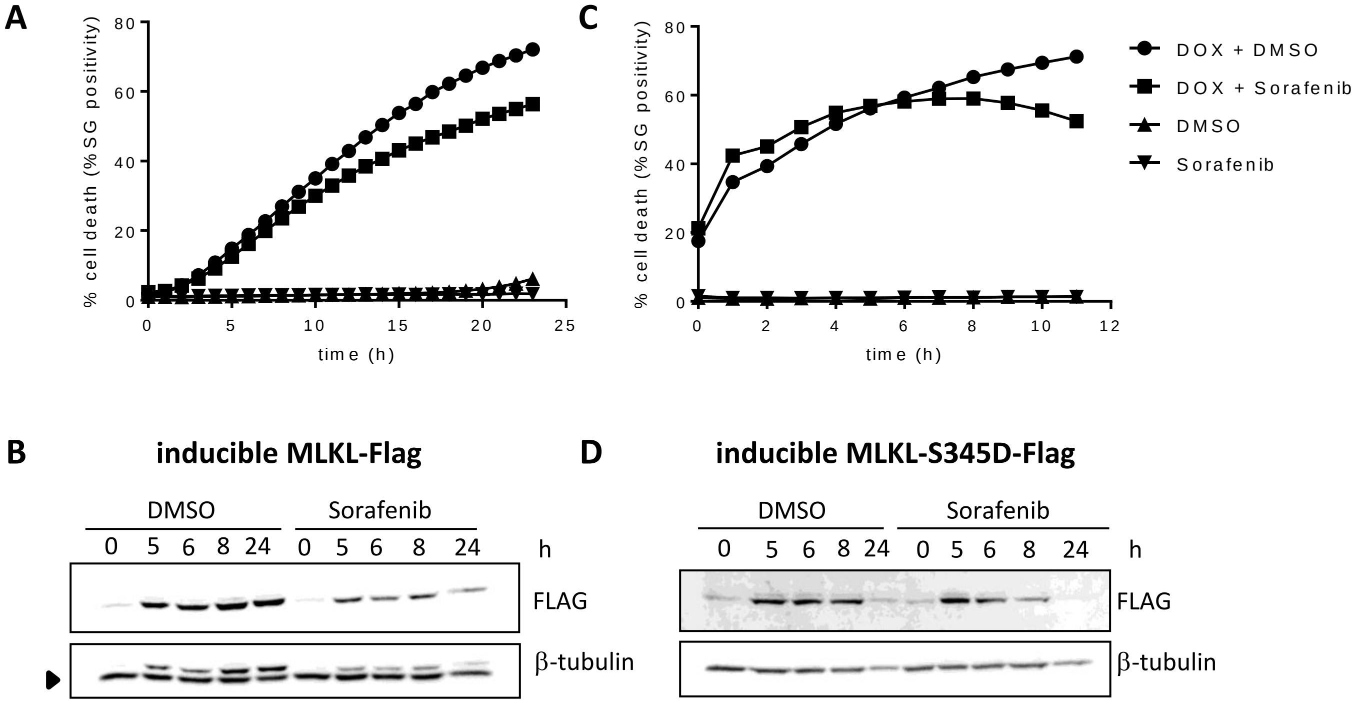

Supplementary Figures

Supplementary Figure S11.

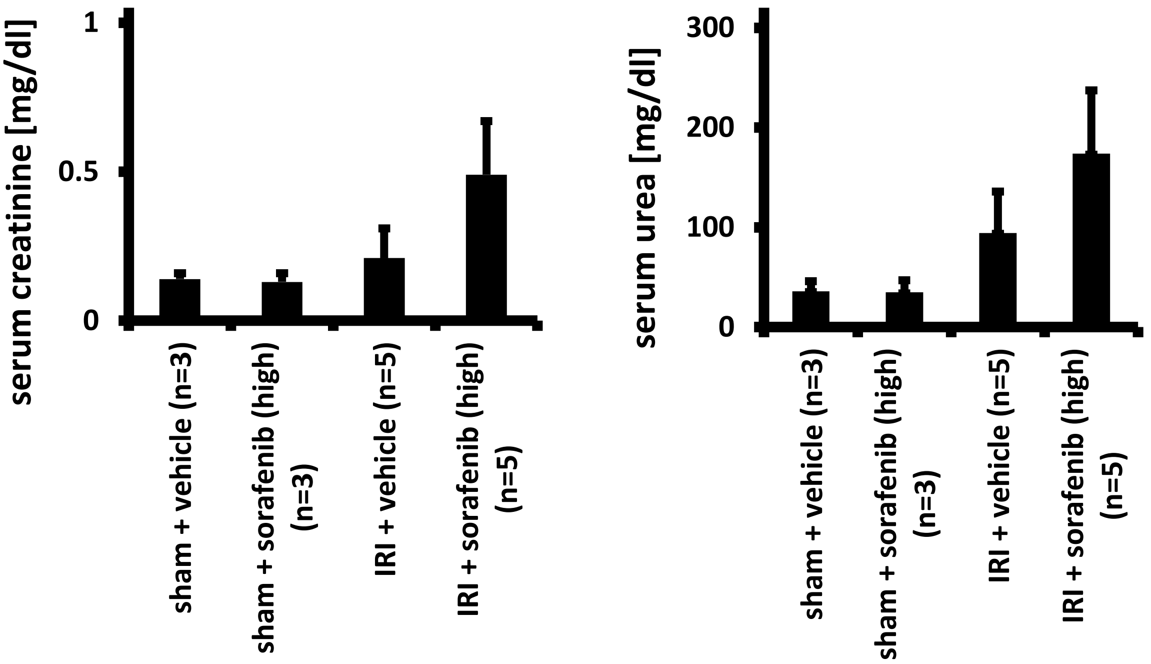

Target family	Compound	Known targets	Cell death *TNF (POC)*	Validation screen *IC50 (µM) (TNF)*	Cell death *TNF/Taki (POC)*
Serine/threonine protein kinases					
RAF	Sorafenib tosylate	C-RAF*, B-RAF*, B-rafV600E*, PDGFRβ, VEGFR 1/2/3, c-Kit, FGFR1, RET, Flt3	41.5	3.25	11.8
Mitogen-actived protein kinases (MAP kinases)					
MEK	PD-98059	MEK1*, MEK2*, HPGD	70.9	NA	98.9
	U-0126	MEK1*, MEK2*, AP-1, STK33	69.7	NA	98.7
Receptor tyrosine kinases					
EGFR	AG-494	EGFR (Erb-B2)*, PDGFR, CDK2, POL E, JMJD2E	58.6	32.26	95.8
VEGFR	SU 4312	VEGFR1/2 (Flk1, Flg)*, PDGFR-β, NOS, ALDH1A1	69.6	26.41	96.8
PDGFR	AG-1296	PDGFRβ*, FGFR, c-Kit	77.6	33.24	93.6
	TYRPHOSTIN 9	PDGFR*, CRAC channel, EGFR	41.8	4.45	95.8
Non-receptor tyrosine kinases					
BCR-ABL	Imatinib mesylate	BCR-ABL*, c-Kit, RET, TrkA, MCSF-1R, PDGFRα/β, DDR1, ABL1	63.9	NA	96.1
JAK	AG-490	JAK1/2*, EGFR (Erb-B2)*, POL E, HADH2	74.2	87.27	97.3

Supplementary table 1. A selection of compounds from the screening assay on L929 cells that protect against necroptosis. Table with a selection of compounds (10 µM) from the cellular screening assay on L929sAhFas cells (Figure 1A-B). Compounds (10 µM) with < 80% cell death (POC) were respectively classified as protective compounds. POC = percent of control. * = primary target. Compound targets were identified using PubChem databank and DrugBank databank. Protective compounds were validated by analysis of a dose response and IC50 calculation. NA = not applicable in the condition of this validation assay.

Target family	Compound	Known targets	cell death *TNF (POC)*	cell death *TNF/Taki (POC)*
Serine/threonine protein kinases				
GSK	Kenpaullone	GSK-3β*, CDK1/Cyclin B, CDK2/cyclin A; E, CDK5/p25, c-Src, CK2, ERK1/2, Lck	162.2	99.9
Mitogen-actived protein kinases (MAP kinases)				
ERK	5-Iodotubercidin	ERK2*, PKA, ADK, CSNK1A1 & CSNK2A1, IRK	228.6	100.9
Receptor tyrosine kinases				
PDGFR	SU11652	PDGFRβ*, Flk-1(VEGFR2), FGFR1, Kit family members, EGFR	194.4	104.0
Non-receptor tyrosine kinases				
Src	PP2	P56lck*, p59fynT*, Hck*, c-Src*, TGF-β1R, CSK, EGFR	127.4	100.2

Supplementary table 2. A selection of compounds from the screening assay on L929 cells that sensitize for necroptosis. Table with a selection of compounds from the cellular screening assay on L929sAhFas cells (FIgure 1A-B). Compounds with > 120% cell death (POC) were classified as sensitizing compounds. POC = percent of control. * = primary target. Compound targets were identified using PubChem databank and DrugBank databank.

Target family	Compound	Known targets	cell death *TNF (POC)*	cell death *TNF/Taki (POC)*
Serine/threonine protein kinases				
PI3K	3-Methyladenine	class I & II & III PI3K* (a.o. Vps34)	88.7	101.5
	LY 294002	PI3Kα,β,δ*, CK2, PIM1, BET	85.7	99.2
	Wortmannin	PI3K*, PI4K, DNA-PK, ATM, MLCK, ATR	99.4	100.8
	Quercetin·2H2O	PI3K*, PIPK, F-ATPase, cAMP & cGMP PDE, PKC, activator of SIRT1, FAS, RECQ1, HADH2	90.4	98.5
Akt/PKB	Triciribine	Akt-1/2/3*, DNA synthesis inhibition, HIV-1	83.6	98.0
	BML-257	AKT1 translocation inhibitor*, HCV NS5B RdRp	91.3	99.7
GSK	Indirubin	GSK-3β*, CDK1/Cyclin B, CDK2/cyclin A;E, CDK4/cyclin D1, CDK5/p35, AHR ligand	91.7	99.1
IRAK	AG-126	IRAK*, ALDH1A1	94.7	99.4
MLCK	ML-7·HCl	MLCK*, PKC, PKA	90.2	99.4
	ML-9·HCl	MLCK*, PKA, PKC, hGEM, hPIM1	84.8	99.8
ROCK	Rockout	ROCK1*, ROCK2*, PRK2, MSK-1, PKA	106.0	99.0
	Y-27632·2HCl	ROCK1 (p160ROCK)*, ROCK2, Prkce, PKC, PKA, PRK2	93.3	98.6
Mitogen-actived protein kinases (MAP kinases)				
p38	SB-202190	p38α *, p38β*, EGFR	110.1	96.3
	SB-203580	p38α/β/β2*, SAPK3/4, RAF1, JNK2-α1-2/β1-2, ALK 5	107.6	99.6
RAF	GW 5074	c-Raf1*, CDK1/2, c-src, ERK2, MEK, p38, Tie2, VEGFR2, c-fms	90.0	99.1
	ZM 336372	c-Raf*, B-RAF	81.9	99.4
Receptor tyrosine kinases				
EGFR	Tyrphostin AG112	EGFR*, PLK1/3, ULK3, STK17A(DRAK1), MNK1, MEKK3, MNK2, BMX(ETK)	89.4	98.7
	BML-265	EGFRK*	118.4	99.6
	Erbstatin analog	EGFRK*	83.3	99.4
	Lavendustin A	EGFRK*, $p60^{c\text{-}src}$, $p56^{lck}$, c-erb B-2, PKA/C	85.9	100.0
	RG-14620	EGFRK*	95.9	99.8
	TYRPHOSTIN 23	EGFRK*, L3MBTL1, ALDH1A1, JMJD2E	88.9	100.0
	TYRPHOSTIN 25	EGFRK*, HADH2, POLK, JMJD2E	90.0	100.0
	TYRPHOSTIN 46	EGFRK*, $p56^{lck}$, PDGFR, POL K, HADH2, JMJD2E	97.8	99.8
	TYRPHOSTIN 47	EGFRK*, PDGFR, $p210^{bcr\text{-}abl}$, POL B/E/I, ALDH1A1, JMJD2E	93.0	100.1
	TYRPHOSTIN 51	EGFRK*	116.5	100.6
	TYRPHOSTIN AG 1478	EGFRK*, MNK1, $p60^{c\text{-}src}$, v-Abl, FBPase	84.2	98.7
	AG-825	HER2*, HER1, PDGFR	80.1	99.4
VEGFR	SU1498	VEGFR2 (Flk1)*, HER2 kinases, PDGFR	91.7	99.2
PDGFR	AG-370	PDGFR*	86.0	99.7
	TYRPHOSTIN AG 1295	PDGFRβ*, VEGFR2, Flt3	93.6	100.1
IRK	HNMPA	IRK*	80.3	100.2
Non-receptor tyrosine kinases				
BTK	LFM-A13	BTK*, Plk1	93.5	100.1
	Terreic acid	BTK*, MurA	103.9	99.1
Src	PP1	P56lck*, p59fynT*, Hck*, c-Src*, TGF-β1R, EGFRK, JAK2, ZAP-70	113.7	100.0
JAK	ZM 449829	JAK3*, EGFR, JAK1, CDK4	87.3	99.6
tyrosine kinases	Genistein	tyrosine kinases*, topoisomerase-II, CLK1, EGFR, hERα/β, hAOX	86.1	99.3
	TYRPHOSTIN AG 1288	tyrosine kinases*, guanylyl cyclase	81.1	99.7

Supplementary table 3. A selection of compounds, from the screening assay on L929 cells, that have no effect on necroptosis. Table with a selection of compounds from the cellular screening assay on L929sAhFas cells (figure 1A-B). Compounds with < 80% cell death (POC) and > 120% cell death (POC) were respectively classified as protective or sensitizing compounds. POC = percent of control. * = primary target. Compound targets were identified using PubChem databank and DrugBank databank.

				IC50 (µM)	
**Species**	**Cell type**	**Pathway**	**Stimulus**	**Sorafenib**	**Nec-1s****
**Murine**	**L929sAhFas**	**Necroptosis**	**mTNF**	1.27 ± 0.90	0.24 ± 0.12
		**Apoptosis**	**Anti-Fas**	-	-
	**MEF**	**Necroptosis**	**mTNF + BV6 + zVAD**	3.48 ± 1.59	0.63 ± 0.25
		**Apoptosis**	**mTNF + BV6**	2.20 ± 0.47*	0.38 ± 0.05*
**Human**	**Jurkat FADD$^{-/-}$**	**Necroptosis**	**hTNF**	14.37 ± 1.85	0.32 ± 0.11
	**HT29**	**Necroptosis**	**hTNF + BV6 + zVAD**	11.51 ± 3.17	0.11 ± 0.02
	**MV4-11**	**Necroptosis**	**BV6 + zVAD**	-	0.15 ± 0.01
	**Molm13**	**Necroptosis**	**BV6 + zVAD**	≤ 0.03	0.28 ± 0.17

Supplementary table 4. Sorafenib inhibits TNF-induced RIPK1-dependent cell death in both murine and human cell lines. Summary table of IC50 values (µM) of figure 2 for inhibition of necroptosis/apoptosis in both murine and human cell lines. Data were normalized to DMSO-treated control cells and represent the mean value ± S.E.M. of three independent experiments (* = duplicates). Toxic concentrations were removed from the analysis. ** = in HT-29 cells, Nec-1 was used in stead of Nec-1s.
